# The low health literacy in Latin America and the Caribbean: a systematic review and meta-analysis

**DOI:** 10.1186/s12889-024-18972-2

**Published:** 2024-06-01

**Authors:** Patricia Romualdo de Jesus, Bianca Vendruscolo Bianchini, Patrícia Klarmann Ziegelmann, Tatiane da Silva Dal Pizzol

**Affiliations:** Postgraduate Program in Epidemiology, Faculty of Medicine, Federal University of Rio Grande do Sul, Porto Alegre, Brazil

**Keywords:** Health literacy, Systematic review, Latin America, Caribbean, Prevalence

## Abstract

**Background:**

Health literacy (HL) impacts people’s health and well-being. In Latin America and the Caribbean (LAC), there are no general estimates of the prevalence of low HL. This study aimed to estimate the prevalence of low HL among citizens of LAC and identify the tools used to measure it.

**Methods:**

We included observational studies quantifying the prevalence of low HL in people living in LAC. We searched PubMed, CINAHL, EMBASE, ERIC, LILACS, PsycINFO, Redalyc, SciELO, Web of Science, PQDT, and the reference lists of the included studies in June 2023. Two reviewers independently conducted the selection, extraction, and risk of bias assessment using the JBI Critical Appraisal Tools. Meta-analysis of proportions using random effects models was used to summarize the prevalence of low HL estimated. This prevalence was measured in each study using different classification methods: word recognition items, reading and numeracy comprehension items, and self-reported comprehension items.

**Results:**

Eighty four studies involving 23,914 participants from 15 countries were included. We identified 23 tools to assess HL, and most of the studies were carried out in health services. The pooled prevalence of low HL were 44.02% (95%CI: 36.12–52.24) for reading and numeracy comprehension items, 50.62% (95%CI: 41.82–59.39) for word recognition items, and 41.73% (95%CI: 31.76–52.43) for self-reported comprehension items.

**Conclusion:**

Despite the variability in the prevalence of low HL and a diversity of tools, the average of low HL is of concern. Almost half of the participants in the included studies have low HL. Most of the studies targeted users of healthcare services. Further research investigating the prevalence of low HL in the general population and actions focused on health education, communication, and information are necessary.

**Trial registration:**

PROSPERO (CRD42021250286).

**Supplementary Information:**

The online version contains supplementary material available at 10.1186/s12889-024-18972-2.

## Background

Health literacy (HL) is the degree to which individuals can find, understand, and use information and services to inform health-related decisions and actions for themselves and others [1]. Many governments recognize its importance, and nations like China and the United States (U.S.) encompass HL in their public health strategies. Healthy China 2030 includes an increase in people’s HL as one of the targets for health promotion [2]. In the U.S., Healthy People 2030 included HL as part of its framework [1] and also signalized that organizations must take action to reduce the complexity of health systems [3].

In Latin America and the Caribbean (LAC), health systems are often uncoordinated and segmented [4], causing difficulties for users in navigating the healthcare system. With an ethnically and culturally diverse population, increasing political instability, and marked levels of inequality [5], exploring this region’s social and health scenario is a great challenge. Socially disadvantaged populations are likelier to have low HL, and several studies suggest that HL may be an explanatory factor in the pathways that generate health disparities [6].

Thus, studies that evaluate the prevalence of low HL and identify the population’s difficulties in finding, understanding, and using health information and navigating healthcare systems are necessary for planning actions to promote health literacy. So far, no systematic reviews have investigated the prevalence of low HL in LAC scenario and its associated factors. Therefore, this systematic review and meta-analysis aimed to estimate the prevalence of low HL in LAC and identify the tools used to measure it.

## Methods

This systematic review was conducted according to the protocol registered in the International Prospective Register of Systematic Reviews (PROSPERO) under CRD42021250286. The reporting follows the Cochrane Handbook recommendations [7] and the Preferred Reporting Items for Systematic Reviews and Meta-Analyses (PRISMA) Statement [8].

### Eligibility criteria

We included: (1) observational studies (i.e., cohort and cross-sectional studies) quantifying the prevalence of low HL in general or specific populations in LAC countries; (2) published and unpublished manuscripts in any language and year of publication. We excluded: (1) studies assessing specific HL (i.e., oral and nutritional HL) (2) studies assessing HL of health professionals and university students, knowledge assessment of health conditions or disease; (3) abstracts, reviews, and protocols; (4) studies that did not report HL prevalence, reporting only the mean scores.

### Information sources and search strategy

We searched PubMed, CINAHL, EMBASE, ERIC, LILACS, PsycINFO, Redalyc, SciELO, and Web of Science databases from inception to June 2023. The grey literature was searched by ProQuest Dissertations and Theses Global (PQDT). References of the included studies were manually screened.

The search strategy was adapted and performed at each database and sources using terms such as “health literacy”, “Latin America”, “Caribbean”, and countries’ names (Additional file 1).

### Selection process

Citations were exported from databases into Rayyan [9] web app to remove duplicates and perform the selection process. Two review authors (PRJ, BVB) independently screened the titles and abstracts and selected the articles for full-text review. A third researcher (TSP) resolved any discrepancies between reviewers. Following a full-text review, the two reviewers (PRJ, BVB) independently determined the final list of included studies, with any discrepancies to be resolved by the third reviewer (TSP).

### Data collection process and data items

Two reviewers (PRJ, BVB) extracted the following data independently: author, title, publication date, country, language, study design, setting, target population, sample size, age, sex, formal education, tools used to assess HL and their validity, and the prevalence of low HL (i.e., the total number of people with low HL among the total number of participants who had their HL level assessed). Disagreements were resolved by consensus, and, if necessary, a third researcher (TSP) was consulted.

The tools were classified based on the assessment method used by Baccolini et al. 2021 [10]: word recognition items, reading or numeracy comprehension items, self-reported comprehension items, or mixed method. We adapted the classification for reading and numeracy comprehension items and did not use the mixed methods classification (involving more than one type of method). This decision was made because the studies included in our review did not align with those categories. Therefore, our studies were categorized as reading and numeracy comprehension items, word recognition items and self-reported comprehension items. Results of the prevalence classified as inadequate, low, and insufficient were considered low HL. Results in intermediate categories such as moderate, marginal, and problematic were not considered low HL. When more than one tool was used to assess the HL, we collected the data from the tool assessing general HL or more than one domain. Study authors were contacted for further information in case of missing or unclear information.

### Study risk of bias assessment

Two reviewers (PRJ, BVB) independently assessed the risk of bias using the Joanna Briggs Institute (JBI) Critical Appraisal Tools - Checklist for studies reporting prevalence data [11]. The tool assesses the risk of bias in the studies’ design, conduct, and analyses. The answer options for all items are Yes, No, Unclear, or Not/Applicable. It consists of 9 items (in parentheses are the necessary information to consider “yes”): 1.sample frame (studies reporting basics information such as sex, age, and education); 2.recruitment of participants (studies using probabilistic sampling); 3.sample size calculation; 4.detail of subjects and setting (enough details to be reproduced by another researcher); 5.sufficient coverage of the sample (few missings); 6.valid methods (studies using validated tools, which means when there is a report assessing their psychometric properties);7. condition measured (researchers/interviewers trained in tool application);8. statistical analysis (effect measure presenting at least confidence interval and p-value); 9.response rate (low rate of refusals). Disagreements between reviewers were resolved by consensus or by a third researcher (TSP).

### Synthesis methods

The pooled prevalence of low HL was calculated by meta-analysis of proportions. First, we pooled all studies according to their countries and the results were shown using the LAC map. Afterward, the prevalence was estimated according to the classification of the assessment method: word recognition items, reading and numeracy comprehension items, and self-reported comprehension items [10].

The meta-analysis were performed using random effects with logit transformation models with 95% confidence interval (CI). The inverse of variance was employed to calculate pooled prevalence. Heterogeneity across studies was estimated using the restricted maximum-likelihood method, with the Q-profile method used for calculating CI. Additionally, a continuity correction of 0.5 was applied in studies with zero cells. Heterogeneity was evaluated by Cochran’s Q test, and the I^2^ statistic was calculated to estimate the percentage of the variation across studies not attributed to sampling error.

Prediction intervals (PI), funnel plots and Begg’s tests for publication bias assessment were calculated whenever possible (i.e., at least three studies for prediction and at least ten studies for funnel plot). All the meta-analyses and the map of the prevalence of low HL were performed using RStudio® version 2023.09.1 with the ‘meta’ and ‘rworldmap’ packages, respectively.

### Secondary analyses

Subgroup meta-analyses were performed considering the target population, tools used to assess HL, tools validation status, country, and setting as potential factors for heterogeneity. The sensitivity analyses was performed by excluding groups that could influence the prevalence estimates of low HL.The subgroups included in the sensitivity analysis were: grey literature, children and adolescents, older people, healthcare service users, and studies from Brazil. Also, the studies with non-validated tools and validation studies were performed in the sensitivity analyses to evaluate the influence of non-validated tools on the prevalence of HL.

## Results

### Study selection

**Fig. 1:**
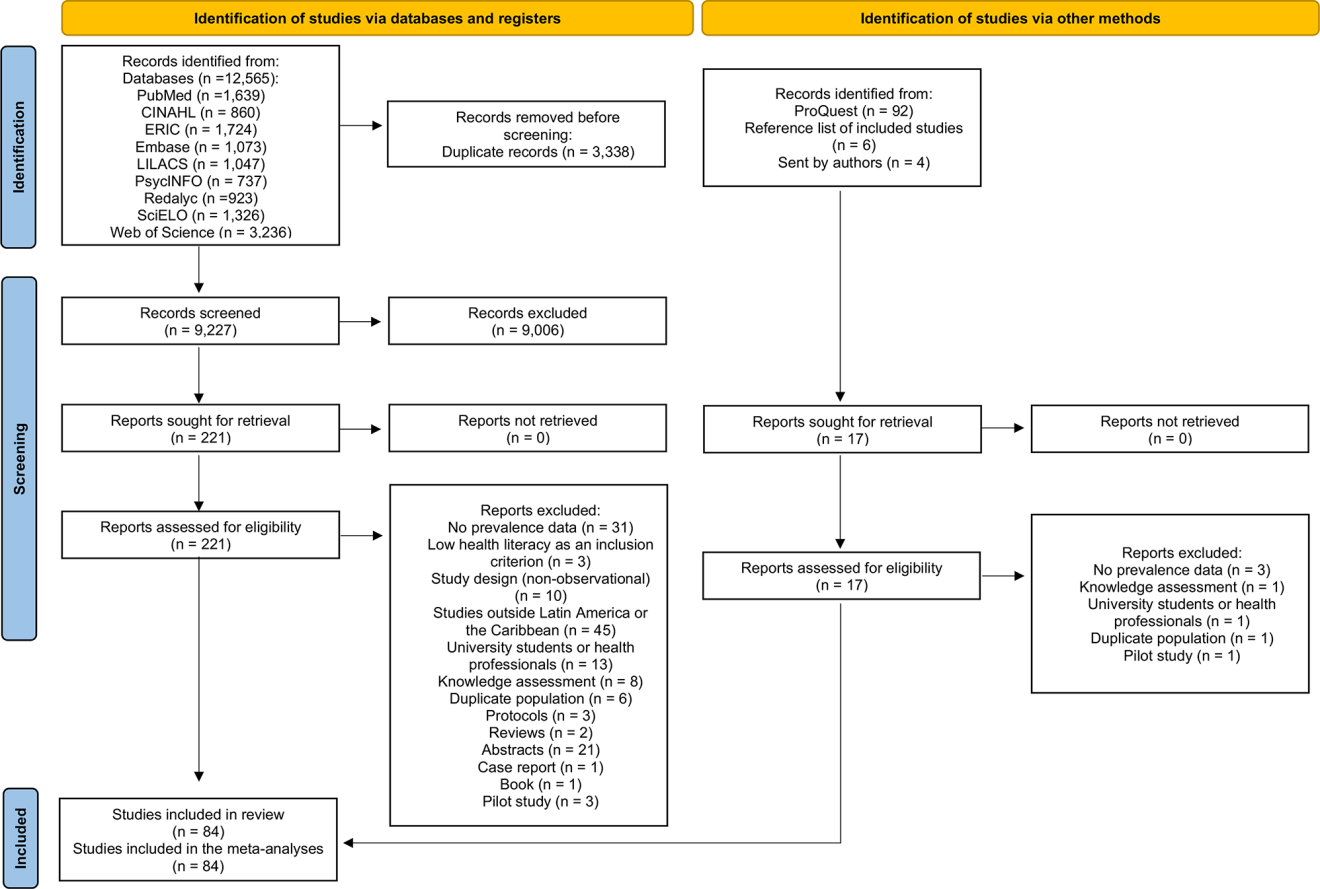
PRISMA flow diagram

After removing duplicates, our search resulted in 9,227 potentially relevant articles (Fig. 1). Following title/abstract screening, 221 full-text articles via databases and 17 via other methods were retrieved and assessed for eligibility, totaling 238 full-text articles. Finally, 74 studies [12–85] from databases and 10 studies [86–95] were identified via other methods, totaling 84 studies. The list of excluded studies and reasons for exclusion are in the supplementary material (Additional file 2).

### Study characteristics

A total of 23,914 people were included in this systematic review (Table 1). Studies were published between 2009 and 2023 and most of them were conducted in Brazil (57 studies; *n* = 11,445; 47.9%) [14–19, 22, 23, 25–34, 41, 42, 45, 49, 50, 52–55, 58–62, 64–66, 68–71, 74–81, 83, 86–94], with a cross-sectional design (82 studies; *n* = 23,660; 98.9%). Most people were interviewed in health services (70 studies; *n* = 14,570; 60.9%) [12–17, 19, 21–23, 25–38, 40, 41, 43–45, 47–67, 69, 71, 73–78, 81–86, 89, 91–95]. The predominant population was general population (15 studies; *n* = 9,112; 38.1%) [18, 20, 22, 24, 26, 35, 41, 44, 52, 63, 64, 71, 72, 80, 85].

**Table 1:** Characteristics of included studies (*n* = 84)

Characteristics	*N* Studies	*N* Population (%)
**Total**	84	23,914 (100)
**Target population**		
General population	15	9,112 (38.1)
Health service users	17	5,313 (22.2)
Hypertensive patients	7	2,198 (9.2)
Diabetic patients	12	1,995 (8.3)
Other pathologies^a^	7	1,718 (7.2)
Older people	8	1,236 (5.2)
Heart disease patients	7	941 (3.9)
Children and adolescents	2	594 (2.5)
HIV patients	2	306 (1.3)
Patients with nephropathies	4	274 (1.1)
Caregivers and parents	3	227 (0.9)
**Sex** ^**b**^		
Female/Male	77	22,735 (95.1)
Female	5	710 (3.0)
Male	1	355 (1.5)
Not reported	1	119 (0.5)
**Year of publication**		
2009–2014	9	1,759 (7.4)
2015–2019	42	9,701 (40.6)
2020–2023	33	12,454 (52.1)
**Country**		
Brazil	57	11,445 (47.9)
Mexico	3	4,912 (20.5)
Puerto Rico	3	2,861 (12.0)
Chile	4	1,242 (5.2)
Peru	5	1,160 (4.9)
Bolivia	1	643 (2.7)
Jamaica	2	443 (1.9)
Argentina	2	385 (1.6)
Guyana	1	228 (1.0)
Guatemala	1	210 (0.9)
Dominican Republic	1	107 (0.4)
Barbados	1	106 (0.4)
Suriname	1	99 (0.4)
Costa Rica	1	51 (0.2)
Honduras	1	22 (0.1)
**Study design**		
Cross-sectional	82	23,660 (98.9)
Cohort	2	254 (1.1)
**Setting**		
Health services	70	14,570 (60.9)
Web-based surveys	4	6,348 (26.5)
Households	7	2,283 (9.5)
Schools	2	594 (2.5)
Not reported	1	119 (0.5)

^a^Chronic conditions, systemic lupus erythematosus, Alzheimer’s Disease, Mild Cognitive Impairment, and hospitalized patients

^b^The total number is higher than the actual value because some studies reported only the total number of participants females and males without specifying how many of each sex were assessed for HL

Due to the different age and formal education presentation formats, the results were shown narratively for studies that reported mean or median. Four studies (*n* = 729; 3.0%) [41, 46, 68, 85] evaluated a population under 30 years of age, and 35 studies (*n* = 8,315; 34.8%) [12, 14, 16, 25, 26, 29, 31, 32, 35, 40, 44, 45, 48, 50, 51, 53, 54, 56, 57, 60, 62–64, 66, 67, 69, 71, 79, 82, 86, 89, 90, 92, 93, 95] assessed HL in adults with an average age between 30 and 60 years of age. 25 studies had a population with an average age above 60 years (*n* = 5,870; 24.5%) [13, 15, 17, 21, 24, 28, 33, 37–39, 42, 49, 55, 59, 61, 65, 70, 75–77, 81, 83, 84, 88, 91]. In the mean years of formal education, 1,849 (7.7%) [13, 16, 26, 29, 40, 54, 69, 85] participants had eight years or more of schooling, and 1,247 (5.2%) [15, 55, 65, 81] had less than eight years of schooling. The table with the individual characteristics of the included studies is in Additional file 3.

We identified 22 tools used to assess HL (Table 2). The most used tool was the the Brazilian portuguese version of Short Test of Functional Health Literacy in Adults (S-TOFHLA) (29 studies; *n* = 5,558; 23.2%) [14, 16, 19, 22, 23, 26, 27, 29, 31, 32, 34, 49, 50, 52, 58, 59, 61, 69, 70, 74–76, 78, 79, 87, 88, 93, 94] followed by Newest Vital Sign (NVS) (7 studies; *n* = 5,632; 23.6%) [20, 43, 44, 46, 64, 71, 91]. Several studies used the term Brief Test of Functional Health Literacy in Adults (B-TOFHLA) [19, 26, 32, 49, 52, 58, 75, 78, 87, 88, 93] referring to the Brazilian version of Short Test of Functional Health Literacy in Adults (S-TOFHLA) [29] that evaluates numeracy and reading comprehension. For standardization, we used the S-TOFHLA nomenclature. Regarding the tools’ validation, most were validated in the country of origin (47 studies; *n* = 9,483; 39.7%) [13, 14, 16, 17, 19, 22, 23, 26–28, 31–34, 38, 39, 41, 46, 49, 50, 54, 55, 58–60, 62, 65–68, 70, 74–81, 83, 84, 86, 88, 91–94]. The predominant language was Portuguese (57 studies; *n* = 11,445; 47.9%) [14–19, 22, 23, 25–34, 41, 42, 45, 49, 50, 52–55, 58–62, 64–66, 68–71, 74–81, 83, 86–94] and Spanish (22 studies; *n* = 11,593; 48.5%) [12, 13, 20, 21, 36–39, 44, 46–48, 51, 57, 63, 67, 72, 73, 82, 84, 85, 95].

**Table 2:** Characteristics of tools in included studies (*n* = 84)

Tools characteristics ^a^	*N* studies	*N* population (%)
**Reading and numeracy comprehension items**		
Brazilian version of Short Test of Functional Health Literacy in Adults (S-TOFHLA)	29	5,558 (23.2)
Newest Vital Sign (NVS)	7	5,632 (23.6)
Test of Functional Health Literacy in Adults (TOFHLA)	1	302 (1.3)
Test of Functional Health Literacy in Adults-Spanish – Puerto Rico version (TOFHLA-SPR)	1	199 (0.8)
**Word recognition items**		
Short Assessment of Health Literacy for Portuguese-speaking Adults (SAHLPA-18)	11	2,289 (9.6)
Short Assessment of Health Literacy for Spanish-speaking Adults (SAHLSA-50)	10	2,515 (10.5)
18-item Short Assessment of Health Literacy Spanish and English (SAHL-S&E)	3	430 (1.8)
Short Assessment of Health Literacy for Portuguese-speaking Adults (SAHLPA-50)	2	289 (1.2)
Rapid Estimate of Adult Literacy in Medicine (REALM)	1	286 (1.2)
Rapid Estimate of Adult Literacy in Medicine–Short Form (REALM-SF)	1	106 (0.4)
Rapid Estimate of Adult Literacy in Medicine adapted for the Dutch language (REALM-D)	1	99 (0.4)
**Self-reported comprehension items**		
14-item Health Literacy Scale (HLS-14)	3	518 (2.2)
Single question	2	2,554 (10.7)
Spanish version of the European Health Literacy questionnaire (HLS-EU-Q47)	2	868 (3.6)
Brief Health Literacy Screening Tool	2	181 (0.8)
Brazilian version of European Health Literacy Survey Questionnaire short-short form (HLS-EU-Q6)	1	783 (3.3)
Ten self-reported questions	1	384 (1.6)
Not reported	1	355 (1.5)
Single Item Literacy Screener (SILS)	1	228 (1.0)
European Health Literacy Survey (HLS-EU-BR)	1	107 (0.4)
Health Literacy Screening Questions (HLSQ)	1	100 (0.4)
Single-item variant of the Subjective Health Literacy Screener (SHLS)	1	98 (0.4)
Eight-Item Health Literacy Assessment Tool	1	33 (0.1)
**Validity**		
Validated in the country	47	9,483 (39.7)
Validated in language but not in the country	15	3,480 (14.6)
Validation study	8	2,568 (10.7)
Not validated in the country of application	8	3,297 (13.8)
Not validated	6	5,086 (21.3)
**Language**		
Portuguese	57	11,445 (47.9)
Spanish^b^	22	11,593 (48.5)
English	4	777 (3.2)
Dutch	1	99 (0.4)

^a^Total number of tools regardless of validation status

^b^Hadden, 2018 and Mora Vicariolli, 2021 also evaluated people who speak English and Cabécar, respectively

### Risk of bias in studies

Table 3 shows the risk of bias assessment. Only 16 studies (*n* = 3,597) used appropriated sampling [14, 18, 21, 22, 24, 40, 43, 47, 51, 54, 63, 68, 80, 83, 86, 89], and 33 studies (*n* = 11,925) had an adequate sample size [13, 14, 18, 20, 22, 23, 28, 30, 31, 35, 38, 43, 47, 48, 50, 54, 62, 68, 69, 73, 74, 79, 80, 83, 84, 86–90, 92–94] (Additional file 6). Half of the participants (*n* = 11.892) were not assessed by a validated tool [20, 21, 30, 35–37, 40, 42–45, 47, 48, 52, 57, 63, 69, 72–74, 82, 85, 87, 89, 95], and the measurement was not measured in a standardized and reliable way for 57.6% of the participants (54 studies; *n* = 13,790) [12, 14, 18–21, 24, 25, 28, 29, 32–34, 36, 39, 40, 42, 43, 45, 47–53, 55, 57–61, 64–66, 68, 69, 73–78, 80–82, 84, 86, 87, 90–93, 95].

**Table 3:** Risk of bias in the included studies

Questions	*N* studies	*N* population (%)
1.The sample frame was appropriate to address the target population.	81	23,332 (97.6)
2. Study participants were sampled appropriately.	16	3,597 (15.0)
3. The sample size was adequate.	33	11,925 (49.9)
4. The study subjects and the setting were described in detail.	80	23,443 (98.0)
5. The data analysis was conducted with sufficient coverage of the identified sample.	69	20,027 (83.7)
6. Valid methods were used to identify the condition.	55	12,022 (50.3)
7. The condition was measured in a standard, reliable way for all participants.	27	7,783 (32.5)
8. There was appropriate statistical analysis.	35	15,513 (64.9)
9. The response rate was adequate, and if not, the low response rate was managed appropriately.	68	19,315 (80.8)

### Results of syntheses

**Fig. 2:**
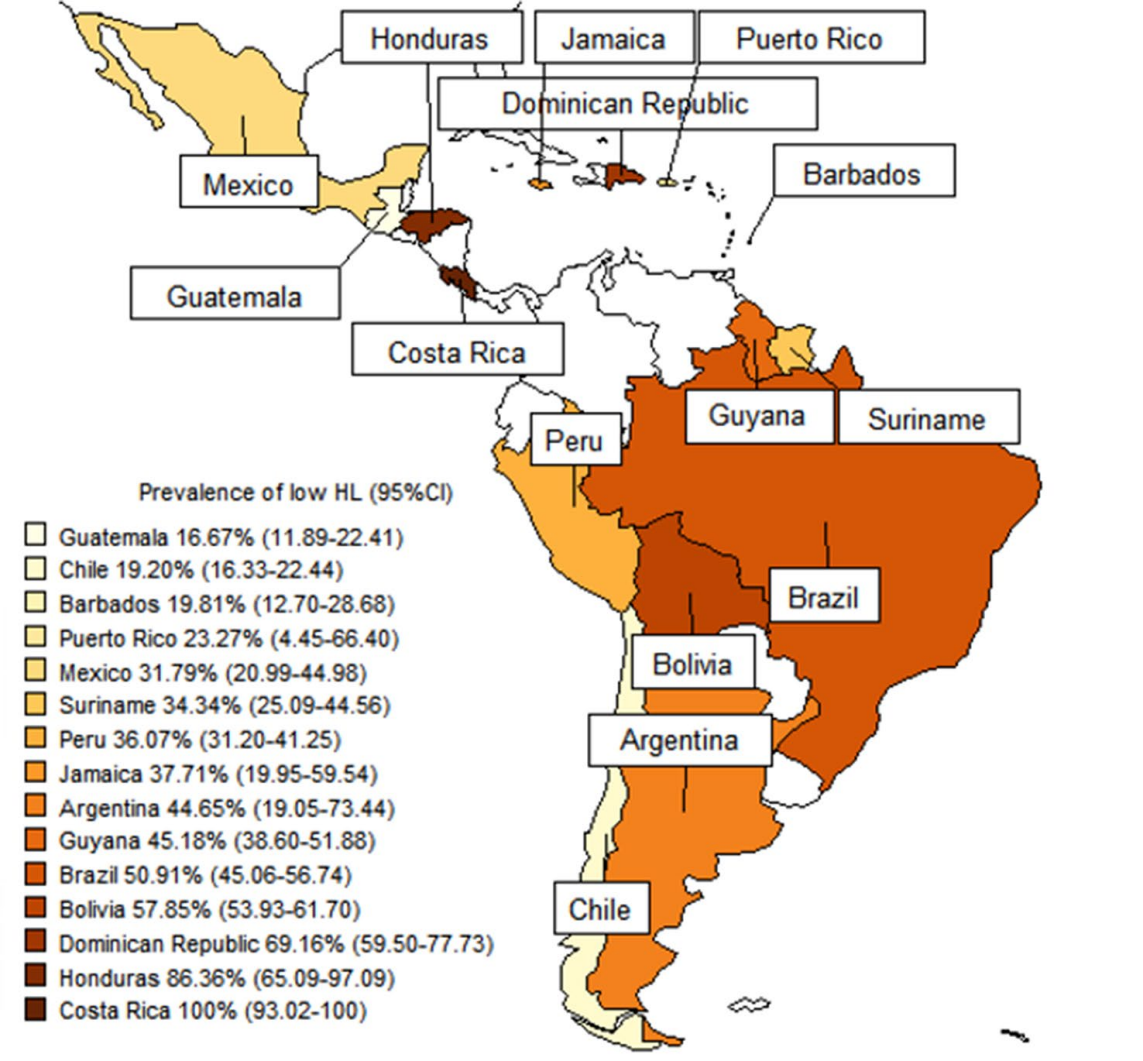
Map with the estimated prevalence of low health literacy in Latin America and the Caribbean according to the country

In total, there are 42 countries in the LAC region [96]. The map shows the estimated prevalence of low HL in 15 countries of this region (Fig. 2). The estimated prevalence of low HL by country were: Argentina varied from 30.13 to 60.26% (44.65%; 95%CI: 19.05–73.44) [21, 47], Brazil varied from 0 to 100% (50.91%; 95%CI: 45.06–56.74; 95%PI: 15.15–85.76) [14–19, 22, 23, 25–34, 41, 42, 45, 49, 50, 52–55, 58–62, 64–66, 68–71, 74–81, 83, 86–94], Chile varied from 20 to 23.53% (19.20%; 95%CI: 16.33–22.44; 95%PI: 10.72–31.98) [13, 38, 39, 84], Jamaica varied from 27.27 to 48.17% (37.71%; 95%CI: 19.95–59.54) [24, 43], Mexico varied from 23.01 to 45.56% (31.79%; 95%CI: 20.99–44.98; 95%PI: 0.04–99.85) [20, 37, 48], Peru varied from 29.04 to 43.00% (36.07%; 95%CI: 31.20-41.25; 95%PI: 21.53–53.71) [51, 63, 73, 85, 95], and Puerto Rico varied from 5.18 to 59.65% (23.27%; 95%CI: 4.45–66.40; 95%PI: 0.00-100.00) [44, 67, 72]. Barbados (19.81%; 95%CI: 12.70-28.68) [40], Bolivia (57.85%; 95%CI: 53.93–61.70) [12]), Costa Rica (100%; 95%CI: 93.02–100) [57], Dominican Republic (69.16%; 95%CI: 59.50-77.73) [82], Guatemala (16.67%; 95%CI: 11.89–22.41) [46], Guyana (45.18%; 95%CI: 38.60-51.88) [56], Honduras (86.36%; 95%CI: 65.09–97.09) [36], and Suriname (34.34%; 95%CI: 25.09–44.56) [35] presented only one study each.

### Low HL according to reading and numeracy comprehension items

**Fig. 3:**
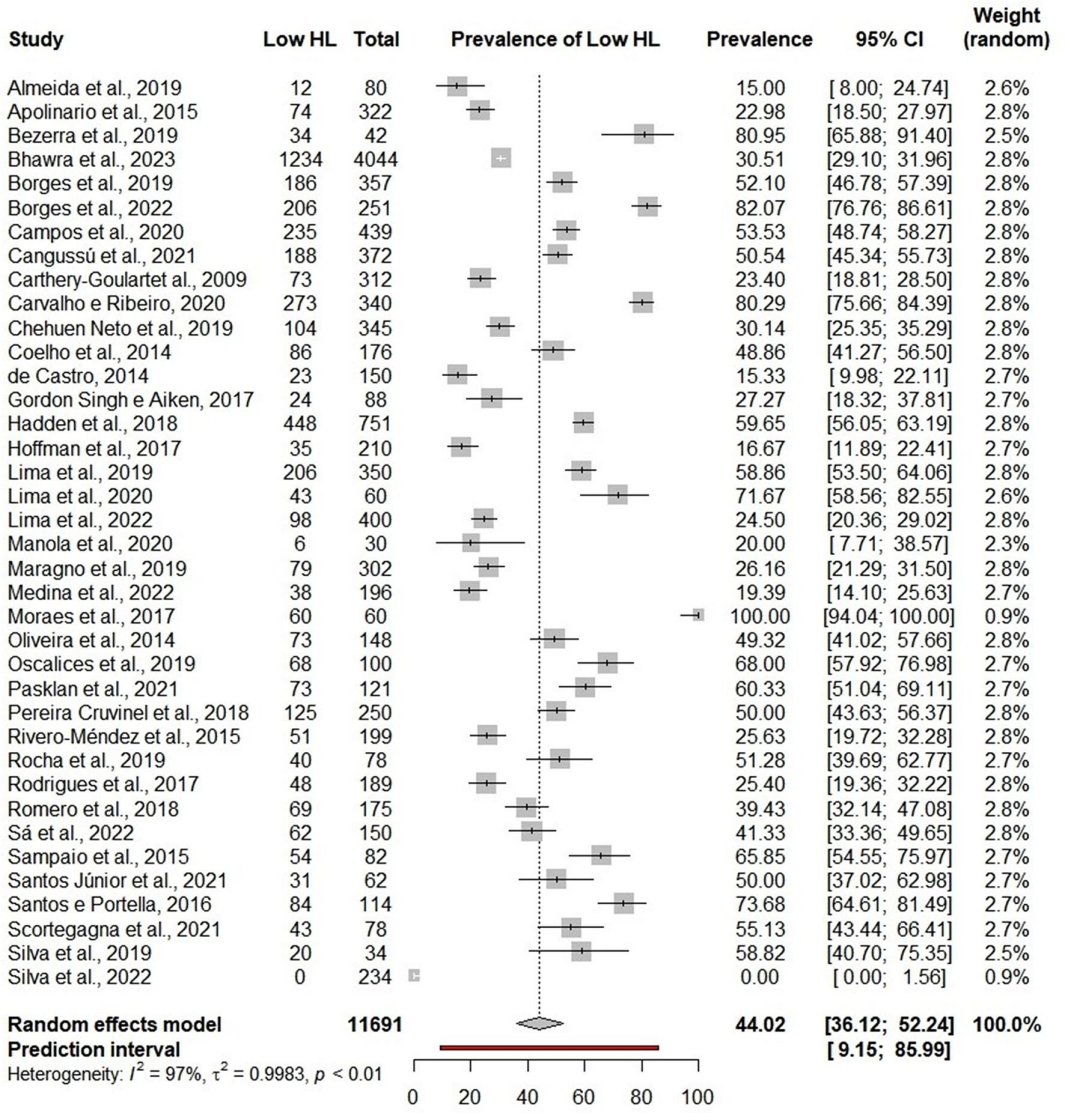
Meta-analysis of low health literacy prevalence in reading and numeracy comprehension items

The overall prevalence of low HL varied considerably from 0 to 100%, with a pooled of 44.02% (95%CI 36.12–52.24; 95%PI: 9.15–85.99; I^2^ = 97%) [14, 16, 19, 20, 22, 23, 26, 27, 29, 31, 32, 34, 43, 44, 46, 49, 50, 52, 53, 58, 59, 61, 64, 67, 69–71, 74–76, 78, 79, 87–89, 91, 93, 94] (Fig. 3). Considering only the studies using the TOFHLA versions, the low HL prevalence (45.40%; 95%CI: 36.09–55.05; 95%PI: 8.46–88.21; I^2^ = 96%) [14, 16, 19, 22, 23, 26, 27, 29, 31, 32, 34, 49, 50, 52, 53, 58, 59, 61, 67, 69, 70, 74–76, 78, 79, 87–89, 93, 94] was higher than those studies that used NVS (38.35%; 95%CI: 24.74–54.06; 95%PI: 5.71–86.46; I^2^ = 98%) [20, 43, 44, 46, 64, 71, 91] there was no statistically significant difference (*p* = 0.45).

In the tools that are not validated in the country of application, (51.28%; 95%CI: 39.69–62.77) [69] higher low HL prevalence was found when compared to the other tools, but without a statistically significant difference (*p* = 0.18). Studies from Brazil (46.28%; 95%CI: 37.46–55.34; 95%PI: 9.36–87.79; I^2^ = 96%) [14, 16, 19, 22, 23, 26, 27, 29, 31, 32, 34, 49, 50, 52, 53, 58, 59, 61, 64, 69–71, 74–76, 78, 79, 87–89, 91, 93, 94] and with patients with nephropathies (86.49%; 95%CI: 40.14–98.39; 95%PI: 0-100; I^2^ = 83%) [19, 58, 78] had a higher prevalence compared to the other subgroups (*p* < 0.01). The results of the subgroup analyses are in Additional file 4.

### Low HL according to word recognition items

**Fig. 4:**
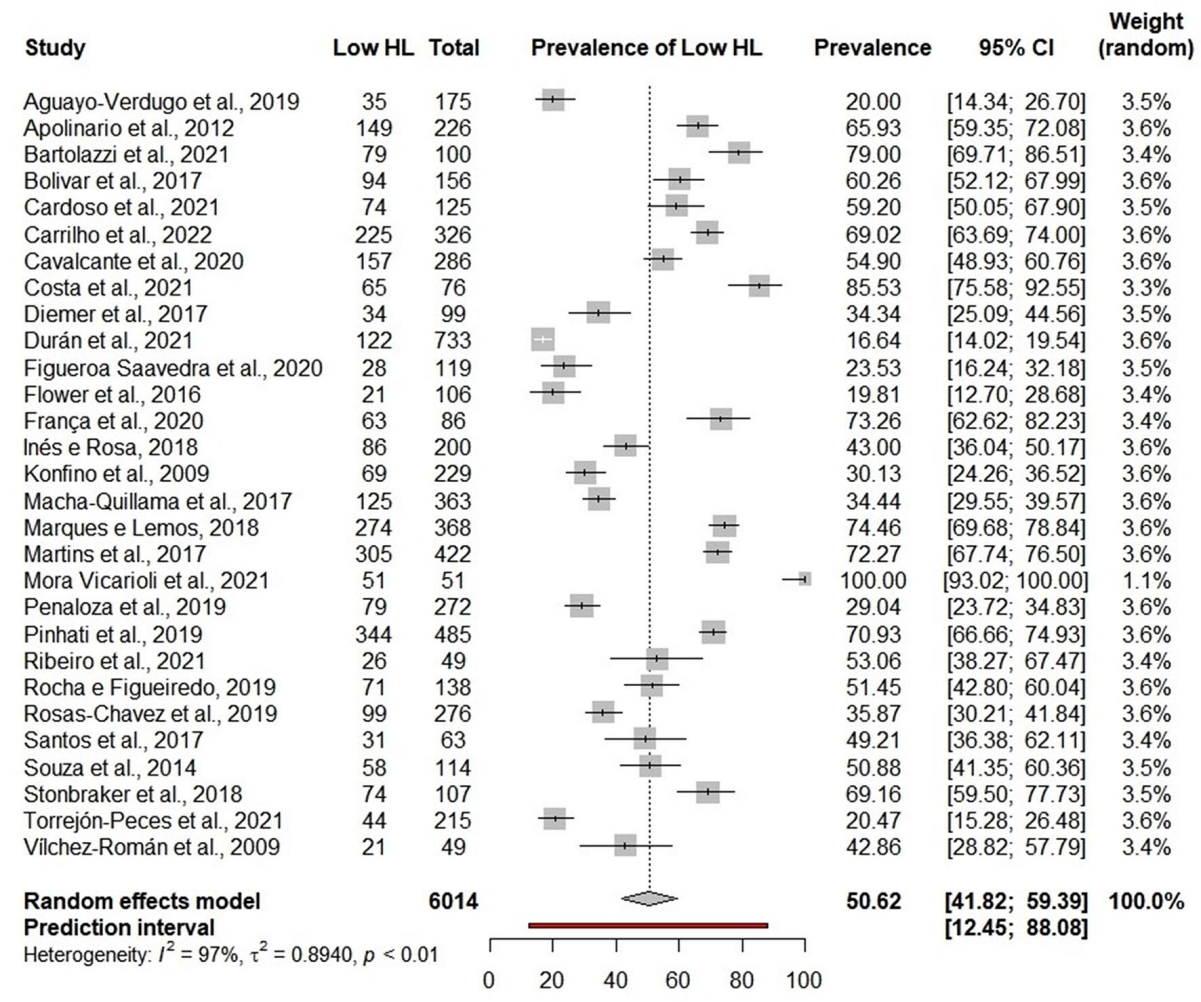
Meta-analysis of low health literacy prevalence in word recognition items

The overall prevalence of low HL varied considerably from 16.64 to 100%, with a pooled of 50.62% (95%CI:41.82–59.39; 95%PI:12.45–88.08; I^2^ = 97%) [13, 15, 17, 21, 28, 30, 33, 35, 38–41, 47, 51, 54, 55, 57, 63, 65, 66, 73, 77, 81, 82, 84–86, 92, 95] (Fig. 4). Subgroup analyses indicated that the tool with higher prevalence of low HL was SAHL-S&E (77.59%; 95%CI: 15.37–98.51; 95%PI: 0-100; I^2^ = 97%) [57, 63, 82] when compared to the other tools (*p* < 0.01).

The tools not validated in the country of application (54.90%; 95%CI: 48.93–60.76) [30] presented higher prevalence of low HL when compared to the other tools, but without a statistically significant difference (*p* = 0.41). Costa Rica (100%; 95%CI: 93.02–100) [57] had the highest prevalence of low HL among the subgroups (*p* < 0.01). Heart disease patients (73.34%; 95%CI: 48.68–88.86) [17, 33, 77] had the highest prevalence compared to the other subgroups but without a statistically significant difference (*p* = 0.13). The results of the subgroup analyses are in Additional file 4.

### Low HL according to self-reported comprehension items

**Fig. 5:**
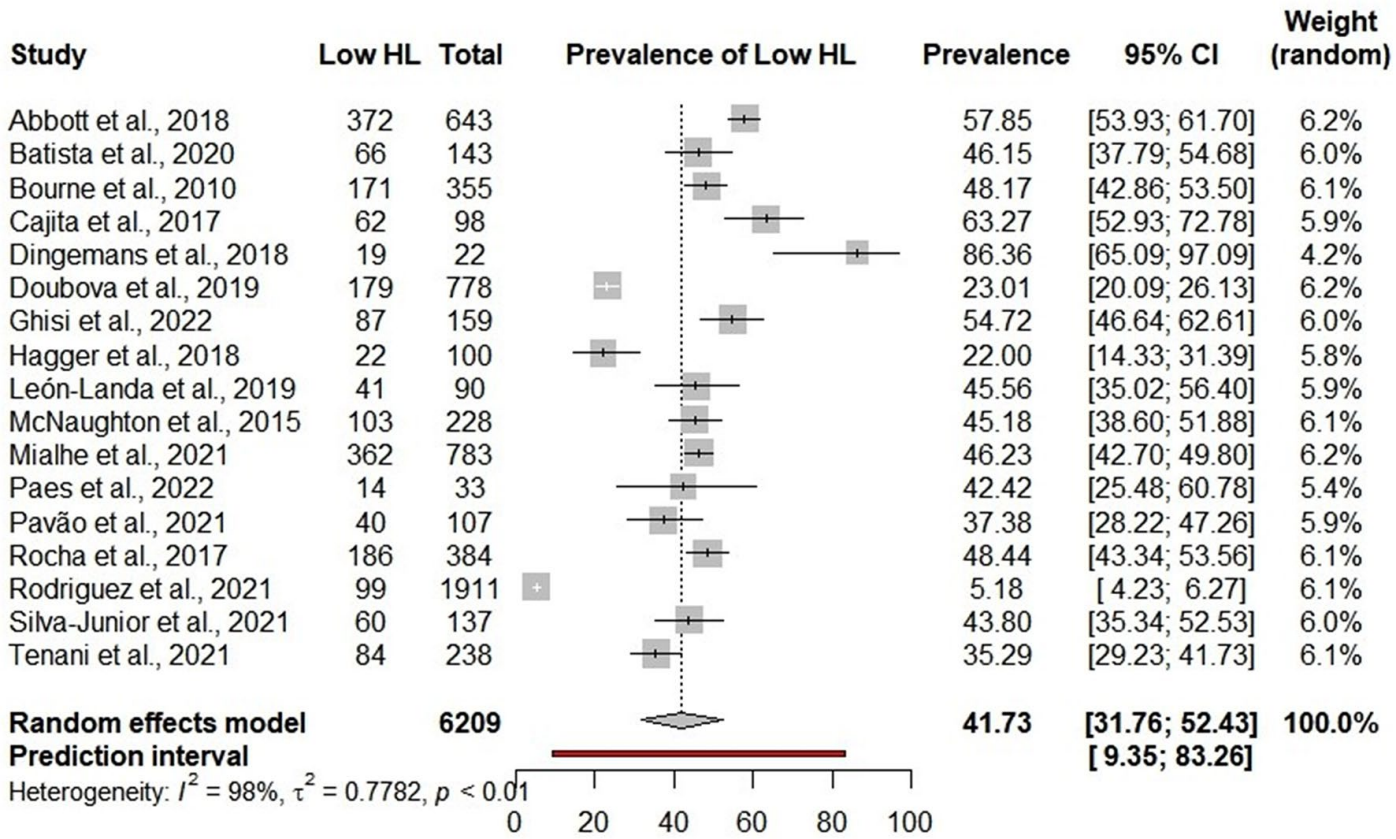
Meta-analysis of low health literacy prevalence in self-reported comprehension items

The overall prevalence of low HL varied considerably from 5.18 to 86.36%, with a pooled of 41.73% (95%CI: 31.76–52.43; 95%PI: 9.35–83.26; I^2^ = 98%) [12, 18, 24, 25, 36, 37, 42, 45, 48, 56, 60, 62, 68, 72, 80, 83, 90] (Fig. 5). Subgroup analyses indicated that the tool that detected a higher prevalence of low HL was the Brief Health Literacy Screening Tool (71.27%; 95%CI: 33.17–92.54; I^2^ = 85%) [36, 42] when compared to the other tools (*p* < 0.01).

Tools that are not validated (48.17%; 95%CI 42.86–53.50) [24] also showed higher prevalence without a statistically significant difference (*p* = 0.42). Honduras (86.36%; 95%CI: 65.09–97.09) [36] had the highest prevalence of low HL among the subgroups (*p* < 0.01). The caregivers and parents (86.36%; 95%CI: 65.09–97.09) [36] had the highest prevalence among the subgroups (*p* < 0.01). The results of the subgroup analyses are in Additional file 4.

### Sensitivity analyses and publication bias

Sensitivity analyses excluding subgroups (grey literature, children and adolescents, older people, healthcare service users, and studies from Brazil) were performed. The results remained similar across all subgroups, except when studies from Brazil were excluded, resulting in a decrease in the prevalence of low HL in reading and numeracy comprehension items, as well as word recognition items. (Additional file 5). The prevalence without tools not validated and validation studies remained similar (reading and numeracy comprehension items, 46.55% [95%CI: 37.17–56.17; PI: 9.41–87.96]; word recognition items, 50.71% [95%CI:41.36–60.01; PI:11.84–88.74]; self-reported comprehension items, 40.79% [95%CI:28.85–53.92; PI:6.91–86.47] (Additional file 7). In the meta-analyses by tools, Begg’s test p-value was not significant (reading and numeracy comprehension items, *p* = 0.9099; word recognition items, *p* = 0.9701; self-reported comprehension items, *p* = 0.3228), with no asymmetry in the funnel plots (Additional file 8).

## Discussion

To our knowledge, this systematic review and meta-analysis summarise, for the first time, the prevalence of low HL in LAC countries. We showed that nearly half of the participants included in the studies presented low HL. Among the three categories of analyzed methods, the estimates found were similar, showing that low HL is an urgent concern for public health in LAC. It was possible to have an overview of which countries are evaluating HL and which tools are being used.

Due to the diversity of tools used in the studies and the lack of a gold standard for HL measuring, we categorized the tools by the assessment method. Word recognition studies evaluated the ability to read and understand commonly used medical terms. The tools using reading and numeracy comprehension items assess the functional ability of the individual to understand texts and use numerical information in everyday situations that occur in healthcare services. Self-reported comprehension items assess other HL domains based on the individual’s self-perception.

Regardless of the assessment method, we found high variability in the prevalence of low LS. Other reviews conducted in the European Union Member States [10], and Southeast Asian [97] have also found high variability in their analyses.

We explored the status validation of the tools and identified that less than half of the studies used tools validated in the language and country of application (39,7%). Some used tools were validated only in the language (14,6%) or not validated in the country of application (13,8%). According to our sensitivity analyses, the use of tools that were not validated did not change the prevalence of low HL.

The process of evaluating psychometric properties influences the accuracy of the measurement, especially in multidimensional constructs such as HL. This aspect is a critical factor, especially in the LAC, since the tools used come from regions with different sociocultural aspects from those of LAC countries. Therefore, translation and cultural adaptation to the local context is fundamental. At this point, we emphasize that although the HL topic is indirectly present in the literature in LAC through other approaches, such as health and patient education, the studies found in this review were published since 2009. Thus, the insertion of HL is a recent movement in LAC countries compared to European and North American countries.

There was a marked disparity in the countries of origin of the studies. Many countries had few studies and most were conducted in Brazil. This discrepancy can be due to the size of the country, which influences the greater number of research centers, universities, researchers, and publications. Based on sensitivity analyses, we can demonstrate that studies originating from Brazil influenced prevalence estimates. These studies increased the prevalence of low HL in reading and numeracy comprehension items, as well as in word recognition items. Furthermore, we must consider that due to the limited number of countries representing the LAC region, there may be insufficient evidence to support the prevalence data. With the map of subgroup analyses of countries, it was possible to show the prevalences of low HL found in the databases. Among the countries with the lowest prevalence of low HL, Guatemala and Barbados had only one study each. We can highlight Chile and Puerto Rico, which had four and three studies respectively, and also showed a low prevalence of low HL. On the other hand, Costa Rica and Honduras, with a high prevalence of low HL, also had only one study found. Hence, we must be cautious when comparing these data, as most countries are underrepresented, and it was not possible to find HL studies from all countries in LAC.

Most studies have evaluated specific populations, focusing mainly on users of health services and populations with some chronic diseases. We observed a decrease in the prevalence of low HL, independent of the evaluation method, in diabetic patients compared to patients with other diseases. We also noticed an increase in low HL in the elderly in the reading and numeracy comprehension items, corroborating with another review [98].

Another point is regarding the setting. Most studies were set in health services, probably due to the financial difficulty of carrying out population-based studies, mainly in LAC.

In general, the quality of the studies was inappropriate. It is essential to perform sample size calculation and random probabilistic sampling to ensure good precision of the summary estimative and population representativeness in prevalence studies [11]. Many studies did not meet these criteria, using convenience samples and not performing sample size calculation. In the question concerning valid methods, we consider valid the tools with some reports demonstrating psychometric properties. Thus, this criterion was met by tools validated in the country and in the validation process. We identified that most tools have flaws in their psychometric properties. Studies evaluating the psychometric properties of tools used to measure HL are necessary to understand these weaknesses [99, 100]. Further research is needed to evaluate the tools used in the LAC scenario.

Overall, most studies used small samples, mainly ones that evaluated specific health conditions or age groups. These findings highlight the lack of research on multicentric studies, such as the European comparative survey [101] and population-based studies in other countries [102–104]. Furthermore, most studies focused on functional HL, assessing reading, numeracy, and comprehension of medical terms. Tools assessing the communicative/interactive HL, which refers to the development of personal skills, and critical HL, which considers more advanced cognitive skills such as individual and community empowerment [105], were few approached.

### Strengths and limitations

This systematic review and meta-analysis brought unprecedented results from a comprehensive search strategy in several databases, including the grey literature, without limitations regarding language, year, and publication status. The methodology was carried out transparently and strictly followed the recommended guidelines for systematic reviews. On the other hand, our review has some limitations that should be considered when interpreting the results. First, studies reporting the mean HL were not included in the review. Second, there was a high heterogeneity and wide prediction intervals in the meta-analysis attributed to methodological differences from observational studies. Third, the low HL used to estimate the prevalence was considered according to the classification of study authors. The high, medium, and low HL estimates are not standardized and vary by study or tool. Intermediate estimates were not considered low HL so that the prevalence may be underestimated.

## Conclusion

This systematic review and meta-analysis showed that almost half of the participants of the studies conducted in LAC countries had low HL. We found several tools assessing HL and a growing interest in the field. Thus far, the studies found had a small sample size and focused on specific populations. National and multicentric studies applying validated tools are needed to identify the profile and needs of this population. Furthermore, the presented estimates are relevant to alert public governance about this critical concern and demand interventions and public policies to improve the people’s HL. Therefore, it is essential to expand the debate on HL in LAC, strengthening health education, information, and communication actions. Multisectoral efforts and actions are needed to empower and increase the HL of LAC citizens.

### Electronic supplementary material

Below is the link to the electronic supplementary material.

Supplementary Material 1

Supplementary Material 2

Supplementary Material 3

Supplementary Material 4

Supplementary Material 5

Supplementary Material 6

Supplementary Material 7

Supplementary Material 8

## Data Availability

Data is provided within the manuscript or supplementary information files.

## Abbreviations

CI: Confidence Interval
HLS-14: 14-item Health Literacy Scale
HLS-EU-BR: European Health Literacy Survey
HLS-EU-Q47: Spanish version of the European Health Literacy Questionnaire
HLS-EU-Q6: Brazilian version of European Health Literacy Survey Questionnaire short-Short Form
HLSQ: Health Literacy Screening Questions
HL: Health Literacy
JBI: Joanna Briggs Institute
LAC: Latin America and the Caribbean
NVS: Newest Vital Sign
PI: Prediction Interval
PRISMA: Preferred Reporting Items for Systematic Reviews and Meta-Analyses
PROSPERO: Prospective Register of Systematic Reviews
PQDT: ProQuest Dissertations and Theses Global
REALM: Rapid Estimate of Adult Literacy in Medicine
REALM-D: Rapid Estimate of Adult Literacy in Medicine Adapted for the Dutch language
REALM-SF: Rapid Estimate of Adult Literacy in Medicine–Short Form
SAHLPA-18: Short Assessment of Health Literacy for Portuguese-speaking Adults
SAHLPA-50: Short Assessment of Health Literacy for Portuguese-speaking Adults
SAHLSA-50: Short Assessment of Health Literacy for Spanish-speaking Adults
SAHL-S&E: 18-item Short Assessment of Health Literacy Spanish and English
SHLS: Single-item variant of the Subjective Health Literacy Screener
S-TOFHLA: Brazilian version of Short Test of Functional Health Literacy in Adults
TOFHLA: Test of Functional Health Literacy in Adults
TOFHLA-SPR: Test of Functional Health Literacy in Adults-Spanish – Puerto Rico version
US: United States
SILS: Single Item Literacy Screener
