# Supplementary material for: The low health literacy in Latin America and the Caribbean: a systematic review and meta-analysis
Source: BMC Public Health. 2024 Jun 1;24:1478. doi: 10.1186/s12889-024-18972-2 (PMC11144327; doi:10.1186/s12889-024-18972-2)
Supplement: Supplementary file 1 — Supplementary Material 1 [file 12889_2024_18972_MOESM1_ESM.pdf]

Additional file 1. Search strategy and keywords.

Database	Search strategy and keywords	Results
PubMed 19/06/23	(Patient Medication Knowledge[mh] OR Patient Medication Knowledge[tw] OR Patient drug knowledge[tw] OR Consumer Health Information[mh] OR Consumer Health Information[tw] OR Literacy[mh] OR literacy[tw] OR Illitera*[tw] OR Health Literacy[mh] OR Health Literacy[tw] OR limited health literacy[tw] OR poor health literacy[tw] OR inadequate health literacy[tw] OR low health literacy[tw] OR adequate health literacy[tw])<br>AND<br>(Latin America[mh] OR Latin America*[tw] OR Caribbean Region*[mh] OR Caribbean*[tw] OR Central America[mh] OR Central America*[tw] OR South America[mh] OR South America*[tw] OR Aruba[mh] OR Aruba*[tw] OR Caribbean Netherlands[mh] OR Caribbean Netherlands*[tw] OR Curacao[mh] OR Curacao*[tw] OR Sint Maarten[mh] Sint Maarten*[tw] OR West Indies[mh] OR West Indies*[tw] OR Antigua and Barbuda[mh] OR Antigua and Barbuda*[tw] OR Bahamas[mh] OR Bahamas*[tw] OR Barbados[mh] OR Barbados*[tw] OR British Virgin Islands[mh] OR British Virgin Islands*[tw] OR Cuba[mh] OR Cuba*[tw] OR Dominica[mh] OR Dominica*[tw] OR Dominican Republic[mh] OR Dominican Republic*[tw] OR Grenada[mh] OR Grenada*[tw] OR Guadeloupe[mh] OR Guadeloupe*[tw] OR Haiti[mh] OR Haiti*[tw] OR Jamaica[mh] OR Jamaica*[tw] OR Martinique[mh] OR Martinique*[tw] OR Puerto Rico[mh] OR Puerto Rico*[tw] OR Saint Kitts and Nevis[mh] OR Saint Kitts and Nevis*[tw] OR Saint Lucia[mh] OR Saint Lucia*[tw] OR Saint Vincent and the Grenadines[mh] OR Saint Vincent and the Grenadines*[tw] OR Trinidad and Tobago[mh] OR Trinidad and Tobago*[tw] OR United States Virgin Islands[mh] OR United States Virgin Islands*[tw] OR Belize[mh] OR Belize*[tw] OR Costa Rica[mh] OR Costa Rica*[tw] OR El Salvador[mh] OR El Salvador*[tw] OR Guatemala[mh] OR Guatemala*[tw] OR Honduras[mh] OR Honduras*[tw] OR Nicaragua[mh] OR Nicaragua*[tw] OR Mexico[mh] OR Mexico*[tw] OR Panama[mh] OR Panama*[tw] OR Argentina[mh] OR Argentina*[tw] OR Bolivia[mh] OR Bolivia*[tw] OR Brazil[mh] OR Brazil*[tw] OR Chile[mh] OR Chile*[tw] OR Colombia[mh] OR Colombia*[tw] OR Ecuador[mh] OR Ecuador*[tw] OR French Guiana[mh] OR French Guiana*[tw] OR Guyana[mh] OR Guyana*[tw] OR Paraguay[mh] OR Paraguay*[tw] OR Peru[mh] OR Peru*[tw] OR Suriname[mh] OR Suriname*[tw] OR Uruguay[mh] OR Uruguay*[tw] OR Venezuela[mh] OR Venezuela*[tw])	1639
CINAHL 19/06/23	(MW("Patient Medication Knowledge" OR "Consumer Health Information" OR Literacy OR "Health Literacy") OR TI("Patient Medication Knowledge" OR "Patient drug knowledge" OR "Consumer Health Information" OR literacy OR "Illitera*" OR "Health Literacy" OR "Alfabetiza*") OR AB("Patient Medication	860

	Knowledge" OR "Patient drug knowledge" OR "Consumer Health Information" OR literacy OR "Illitera*" OR "Health Literacy" OR "Alfabetiza*")) AND (MH("Latin America" OR "Caribbean Region*" OR "Central America" OR "South America" OR Aruba OR "Caribbean Netherlands" OR Curacao OR "Sint Maarten" OR "West Indies" OR "Antigua and Barbuda" OR Bahamas OR Barbados OR "British Virgin Islands" OR Cuba OR Dominica OR "Dominican Republic" OR Grenada OR Guadeloupe OR Haiti OR Jamaica OR Martinique OR "Puerto Rico" OR "Saint Kitts and Nevis" OR "Saint Lucia" OR "Saint Vincent and the Grenadines" OR "Trinidad and Tobago" OR "United States Virgin Islands" OR Belize OR "Costa Rica" OR "El Salvador" OR Guatemala OR Honduras OR Nicaragua OR Mexico OR Panama OR Argentina OR Bolivia OR Brazil OR Chile OR Colombia OR Ecuador OR "French Guiana" OR Guyana OR Paraguay OR Peru OR Suriname OR Uruguay OR Venezuela) OR TI("Latin America" OR "Caribbean Region*" OR "Central America" OR "South America" OR Aruba OR "Caribbean Netherlands" OR Curacao OR "Sint Maarten" OR "West Indies" OR "Antigua and Barbuda" OR Bahamas OR Barbados OR "British Virgin Islands" OR Cuba OR Dominica OR "Dominican Republic" OR Grenada OR Guadeloupe OR Haiti OR Jamaica OR Martinique OR "Puerto Rico" OR "Saint Kitts and Nevis" OR "Saint Lucia" OR "Saint Vincent and the Grenadines" OR "Trinidad and Tobago" OR "United States Virgin Islands" OR Belize OR "Costa Rica" OR "El Salvador" OR Guatemala OR Honduras OR Nicaragua OR Mexico OR Panama OR Argentina OR Bolivia OR Brazil OR Chile OR Colombia OR Ecuador OR "French Guiana" OR Guyana OR Paraguay OR Peru OR Suriname OR Uruguay OR Venezuela) OR AB("Latin America" OR "Caribbean Region*" OR "Central America" OR "South America" OR Aruba OR "Caribbean Netherlands" OR Curacao OR "Sint Maarten" OR "West Indies" OR "Antigua and Barbuda" OR Bahamas OR Barbados OR "British Virgin Islands" OR Cuba OR Dominica OR "Dominican Republic" OR Grenada OR Guadeloupe OR Haiti OR Jamaica OR Martinique OR "Puerto Rico" OR "Saint Kitts and Nevis" OR "Saint Lucia" OR "Saint Vincent and the Grenadines" OR "Trinidad and Tobago" OR "United States Virgin Islands" OR Belize OR "Costa Rica" OR "El Salvador" OR Guatemala OR Honduras OR Nicaragua OR Mexico OR Panama OR Argentina OR Bolivia OR Brazil OR Chile OR Colombia OR Ecuador OR "French Guiana" OR Guyana OR Paraguay OR Peru OR Suriname OR Uruguay OR Venezuela))	
ERIC 19/06/23	("Patient Medication Knowledge" OR "Patient drug knowledge" OR "Consumer Health Information" OR Literacy OR Illitera* OR "Health Literacy" OR Alfabetiza*) AND ("Latin America" OR "Caribbean Region" OR Caribbean* OR "Central America" OR "South America" OR Aruba OR "Caribbean Netherlands" OR Curacao OR "Sint Maarten" OR "West Indies" OR "Antigua and Barbuda" OR Bahamas OR	1724

	Barbados OR "British Virgin Islands" OR Cuba OR Dominica OR "Dominican Republic" OR Grenada OR Guadeloupe OR Haiti OR Jamaica OR Martinique OR "Puerto Rico" OR "Saint Kitts and Nevis" OR "Saint Lucia" OR "Saint Vincent and the Grenadines" OR "Trinidad and Tobago" OR "United States Virgin Islands" OR Belize OR "Costa Rica" OR "El Salvador" OR Guatemala OR Honduras OR Nicaragua OR Mexico OR Panama OR Argentina OR Bolivia OR Brazil OR Chile OR Colombia OR Ecuador OR "French Guiana" OR Guyana OR Paraguay OR Peru OR Suriname OR Uruguay OR Venezuela)	
Embase 19/06/23	('Patient education'/exp OR 'Patient Medication Knowledge':ti,ab,kw OR 'Patient drug knowledge':ti,ab,kw OR 'Consumer Health Information'/exp OR 'Consumer Health Information':ti,ab,kw OR Literacy/exp OR literacy:ti,ab,kw OR Illitera*:ti,ab,kw OR 'Health Literacy'/exp OR 'Health Literacy':ti,ab,kw OR 'limited health literacy':ti,ab,kw OR 'poor health literacy':ti,ab,kw OR 'inadequate health literacy':ti,ab,kw OR 'low health literacy':ti,ab,kw OR 'adequate health literacy':ti,ab,kw)<br>AND<br>('South and Central America'/exp OR 'Latin America*':ti,ab,kw OR Caribbean/exp OR Caribbean*:ti,ab,kw OR 'Central America'/exp OR 'Central America*':ti,ab,kw OR 'South America'/exp OR 'South America*':ti,ab,kw OR Aruba/exp OR Aruba*:ti,ab,kw OR 'Caribbean Netherlands'/exp OR 'Caribbean netherlands*':ti,ab,kw OR Curacao/exp OR Curacao*:ti,ab,kw OR 'Saint Martin'/exp OR 'Sint Maarten*':ti,ab,kw OR 'Caribbean Islands'/exp OR 'West Indies*':ti,ab,kw OR 'Antigua and Barbuda'/exp OR 'Antigua and Barbuda*':ti,ab,kw OR Bahamas/exp OR Bahamas*:ti,ab,kw OR Barbados/exp OR Barbados*:ti,ab,kw OR 'British Virgin Islands'/exp OR 'British Virgin Islands*':ti,ab,kw OR Cuba/exp OR Cuba*:ti,ab,kw OR Dominica/exp OR Dominica*:ti,ab,kw OR 'Dominican Republic'/exp OR 'Dominican Republic*':ti,ab,kw OR Grenada/exp OR Grenada*:ti,ab,kw OR Guadeloupe/exp OR Guadeloupe*:ti,ab,kw OR Haiti/exp OR Haiti*:ti,ab,kw OR Jamaica/exp OR Jamaica*:ti,ab,kw OR Martinique/exp OR Martinique*:ti,ab,kw OR 'Puerto Rico'/exp OR 'Puerto Rico*':ti,ab,kw OR 'Saint Kitts and Nevis'/exp OR 'Saint Kitts and Nevis*':ti,ab,kw OR 'Saint Lucia'/exp OR 'Saint Lucia*':ti,ab,kw OR 'Saint Vincent and the Grenadines'/exp OR 'Saint Vincent and the Grenadines*':ti,ab,kw OR 'Trinidad and Tobago'/exp OR 'Trinidad and Tobago*':ti,ab,kw OR 'Virgin Islands'/exp OR 'United States Virgin Islands*':ti,ab,kw OR Belize/exp OR Belize*:ti,ab,kw OR 'Costa Rica'/exp OR 'Costa Rica*':ti,ab,kw OR 'El Salvador'/exp OR 'El Salvador*':ti,ab,kw OR Guatemala/exp OR Guatemala*:ti,ab,kw OR Honduras/exp OR Honduras*:ti,ab,kw OR Nicaragua/exp OR Nicaragua*:ti,ab,kw OR Mexico/exp OR Mexico*:ti,ab,kw OR Panama/exp OR Panama*:ti,ab,kw OR Argentina/exp OR Argentina*:ti,ab,kw OR Bolivia/exp OR Bolivia*:ti,ab,kw OR Brazil/exp OR Brazil*:ti,ab,kw OR Chile/exp OR Chile*:ti,ab,kw OR Colombia/exp OR Colombia*:ti,ab,kw OR Ecuador/exp OR	1073

	Ecuador*:ti,ab,kw OR 'French Guiana'/exp OR 'French Guiana*':ti,ab,kw OR Guyana/exp OR Guyana*:ti,ab,kw OR Paraguay/exp OR Paraguay*:ti,ab,kw OR Peru/exp OR Peru*:ti,ab,kw OR Suriname/exp OR Suriname*:ti,ab,kw OR Uruguay/exp OR Uruguay*:ti,ab,kw OR Venezuela/exp OR Venezuela*:ti,ab,kw)	
LILACS 19/06/23	(mh:(N02.421.726.407.229.500.500* OR I02.233.332.186* OR F01.145.209.429*) OR ti:("Patient Medication Knowledge" OR "Patient drug knowledge" OR "Conhecimento do Paciente sobre a Medicação" OR "Conocimiento de la Medicación por el Paciente" OR "Consumer Health Information" OR "Informação de Saúde ao Consumidor" OR "Información de Salud al Consumidor" OR literacy OR Alfabetiza* OR Illitera* OR "Letramento em saúde" OR "Alfabetización en Salud" OR "literacia en salud" OR "literacia em saúde") OR ab:("Patient Medication Knowledge" OR "Patient drug knowledge" OR "Conhecimento do Paciente sobre a Medicação" OR "Conocimiento de la Medicación por el Paciente" OR "Consumer Health Information" OR "Informação de Saúde ao Consumidor" OR "Información de Salud al Consumidor" OR literacy OR Alfabetiza* OR Illitera* OR "Letramento em saúde" OR "Alfabetización en Salud" OR "literacia en salud" OR "literacia em saúde"))<br>AND<br>(mh:(Z01.107.084* OR Z01.107.169* OR Z01.107.424 OR Z01.107.757*) OR ti:("Latin America" OR "America Latina" OR Caribbe* OR "Central America" OR "America Central" OR Aruba* OR Caribe* OR Curacao* OR Curazao OR "Sint Maarten" OR "São Martinho" OR "West Indies" OR "Índias Ocidentais" OR Antigua OR Barbuda OR Bahamas* OR Barbados* OR "British Virgin Islands" OR Cuba* OR Dominica* OR Grenada* OR Granada OR Guadeloupe* OR Guadalupe OR Haiti* OR Jamaica* OR Martinique* OR Martinica OR "Puerto Rico" OR "Porto Rico" OR "Saint Kitts" OR Nevis OR "São Cristóvão" OR "Saint Kitts" OR "Saint Lucia" OR "Santa Lucia" OR "Saint Vincent" OR Grenadin* OR "São Vicente" OR Granadinas OR "San Vicente" OR Trinidad OR Tobago OR "United States Virgin Islands" OR "Ilhas Virgens Americanas" OR "Islas Virgenes de los Estados Unidos" OR Belize* OR Belice OR "Costa Rica" OR "El Salvador" OR Guatemal* OR Hondur* OR Nicaragu* OR Mexic* OR Panam* OR Argentin* OR Bolivia* OR Brazil* OR Brasil OR Chile* OR Colombia* OR Ecuador* OR Equador OR Guyana* OR Guiana OR Paraguay* OR Paraguai* OR Peru* OR Surinam* OR Uruguai* OR Uruguay* OR Venezuela*) OR ab:("Latin America" OR "America Latina" OR Caribbe* OR "Central America" OR "America Central" OR Aruba* OR Caribe* OR Curacao* OR Curazao OR "Sint Maarten" OR "São Martinho" OR "West Indies" OR "Índias Ocidentais" OR Antigua OR Barbuda OR Bahamas* OR Barbados* OR "British Virgin Islands" OR Cuba* OR Dominica* OR Grenada* OR Granada OR Guadeloupe* OR Guadalupe OR Haiti* OR Jamaica* OR Martinique* OR Martinica OR "Puerto Rico" OR "Porto Rico" OR "Saint Kitts" OR Nevis OR "São Cristóvão" OR "Saint Kitts" OR "Saint	1047

	Lucia" OR "Santa Lucia" OR "Saint Vincent" OR Grenadin* OR "São Vicente" OR Granadinas OR "San Vicente" OR Trinidad OR Tobago OR "United States Virgin Islands" OR "Ilhas Virgens Americanas" OR "Islas Virgenes de los Estados Unidos" OR Belize* OR Belice OR "Costa Rica" OR "El Salvador" OR Guatemal* OR Hondur* OR Nicaragu* OR Mexic* OR Panam* OR Argentin* OR Bolivia* OR Brazil* OR Brasil OR Chile* OR Colombia* OR Ecuador* OR Equador OR Guyana* OR Guiana OR Paraguay* OR Paraguai* OR Peru* OR Surinam* OR Uruguai* OR Uruguay* OR Venezuela*))	
PsycINFO 19/06/23	Index terms:{patient Medication Knowledge} OR {Consumer Health Information} OR {Literacy} OR {Health Literacy} OR<br>Title: ("Patient Medication Knowledge" OR "Patient drug knowledge" OR "Consumer Health Information" OR Literacy OR Illitera* OR "Health Literacy" OR Alfabetiza*) OR<br>Abstract: ("Patient Medication Knowledge" OR "Patient drug knowledge" OR "Consumer Health Information" OR Literacy OR Illitera* OR "Health Literacy" OR Alfabetiza*)<br>AND<br>Index terms:{Latin America} OR {Caribbean Region} OR {Central America} OR {South America} OR {Aruba} OR {Caribbean Netherlands} OR {Curacao} OR {Sint Maarten} OR {West Indies} OR {Antigua and Barbuda} OR {Bahamas} OR {Barbados} OR {British Virgin Islands} OR {Cuba} OR {Dominica} OR {Dominican Republic} OR {Grenada} OR {Guadeloupe} OR {Haiti} OR {Jamaica} OR {Martinique} OR {Puerto Rico} OR {Saint Kitts and Nevis} OR {Saint Lucia} OR {Saint Vincent and the Grenadines} OR {Trinidad and Tobago} OR {United States Virgin Islands} OR {Belize} OR {Costa Rica} OR {El Salvador} OR {Guatemala} OR {Honduras} OR {Nicaragua} OR {Mexico} OR {Panama} OR {Argentina} OR {Bolivia} OR {Brazil} OR {Chile} OR {Colombia} OR {Ecuador} OR {French Guiana} OR {Guyana} OR {Paraguay} OR {Peru} OR {Suriname} OR {Uruguay} OR {Venezuela} OR<br>Title: ("Latin America" OR "Caribbean Region*" OR "Central America" OR "South America" OR Aruba OR "Caribbean Netherlands" OR Curacao OR "Sint Maarten" OR "West Indies" OR "Antigua and Barbuda" OR Bahamas OR Barbados OR "British Virgin Islands" OR Cuba OR Dominica OR "Dominican Republic" OR Grenada OR Guadeloupe OR Haiti OR Jamaica OR Martinique OR "Puerto Rico" OR "Saint Kitts and Nevis" OR "Saint Lucia" OR "Saint Vincent and the Grenadines" OR "Trinidad and Tobago" OR "United States Virgin Islands" OR Belize OR "Costa Rica" OR "El Salvador" OR Guatemala OR Honduras OR Nicaragua OR Mexico OR Panama OR Argentina OR Bolivia OR Brazil OR Chile OR Colombia OR Ecuador OR "French Guiana" OR Guyana OR Paraguay OR Peru OR Suriname OR Uruguay OR Venezuela) OR<br>Abstract: ("Latin America" OR "Caribbean Region*" OR "Central America" OR "South America" OR Aruba OR	737

	"Caribbean Netherlands" OR Curacao OR "Sint Maarten" OR "West Indies" OR "Antigua and Barbuda" OR Bahamas OR Barbados OR "British Virgin Islands" OR Cuba OR Dominica OR "Dominican Republic" OR Grenada OR Guadeloupe OR Haiti OR Jamaica OR Martinique OR "Puerto Rico" OR "Saint Kitts and Nevis" OR "Saint Lucia" OR "Saint Vincent and the Grenadines" OR "Trinidad and Tobago" OR "United States Virgin Islands" OR Belize OR "Costa Rica" OR "El Salvador" OR Guatemala OR Honduras OR Nicaragua OR Mexico OR Panama OR Argentina OR Bolivia OR Brazil OR Chile OR Colombia OR Ecuador OR "French Guiana" OR Guyana OR Paraguay OR Peru OR Suriname OR Uruguay OR Venezuela)	
Redalyc 19/06/23	“Patient Medication Knowledge”, “Patient drug knowledge”, “Consumer Health Information”, “Health Literacy”, “Conocimiento de la Medicación por el Paciente”, “Alfabetización en Salud”, “Información de Salud al Consumidor”, “Literacia en salud”, “Conhecimento do Paciente sobre a Medicação”, “Informação de Saúde ao Consumidor”, “Letramento em saúde” e “Literacia em saúde”.	923
SciELO 19/06/23	("Patient Medication Knowledge" OR "Patient drug knowledge" OR "Consumer Health Information" OR Literacy OR Illitera* OR "Health Literacy" OR Alfabetiza*) AND ("Latin America" OR "Caribbean Region" OR Caribbean* OR "Central America" OR "South America" OR Aruba OR "Caribbean Netherlands" OR Curacao OR "Sint Maarten" OR "West Indies" OR "Antigua and Barbuda" OR Bahamas OR Barbados OR "British Virgin Islands" OR Cuba OR Dominica OR "Dominican Republic" OR Grenada OR Guadeloupe OR Haiti OR Jamaica OR Martinique OR "Puerto Rico" OR "Saint Kitts and Nevis" OR "Saint Lucia" OR "Saint Vincent and the Grenadines" OR "Trinidad and Tobago" OR "United States Virgin Islands" OR Belize OR "Costa Rica" OR "El Salvador" OR Guatemala OR Honduras OR Nicaragua OR Mexico OR Panama OR Argentina OR Bolivia OR Brazil OR Chile OR Colombia OR Ecuador OR "French Guiana" OR Guyana OR Paraguay OR Peru OR Suriname OR Uruguay OR Venezuela)	1326
Web of Science 19/06/23	TS=("Patient Medication Knowledge" OR "Patient drug knowledge" OR "Consumer Health Information" OR Literacy OR Illitera* OR "Health Literacy" OR Alfabetiza*) AND TS=("Latin America" OR "Caribbean Region" OR Caribbean* OR "Central America" OR "South America" OR Aruba OR "Caribbean Netherlands" OR Curacao OR "Sint Maarten" OR "West Indies" OR "Antigua and Barbuda" OR Bahamas OR Barbados OR "British Virgin Islands" OR Cuba OR Dominica OR "Dominican Republic" OR Grenada OR Guadeloupe OR Haiti OR Jamaica OR Martinique OR "Puerto Rico" OR "Saint Kitts and Nevis" OR "Saint Lucia" OR "Saint Vincent and the Grenadines" OR "Trinidad and Tobago" OR "United States Virgin Islands" OR Belize OR "Costa Rica" OR "El Salvador" OR Guatemala OR Honduras OR Nicaragua OR Mexico OR	2886

	Panama OR Argentina OR Bolivia OR Brazil OR Chile OR Colombia OR Ecuador OR "French Guiana" OR Guyana OR Paraguay OR Peru OR Suriname OR Uruguay OR Venezuela)	
Web of Science 19/06/23	TS=("Patient Medication Knowledge" OR "Patient drug knowledge" OR "Consumer Health Information" OR "Health Literacy")<br>AND<br>TS=("Latin America" OR "Caribbean Region" OR Caribbean* OR "Central America" OR "South America" OR Aruba OR "Caribbean Netherlands" OR Curacao OR "Sint Maarten" OR "West Indies" OR "Antigua and Barbuda" OR Bahamas OR Barbados OR "British Virgin Islands" OR Cuba OR Dominica OR "Dominican Republic" OR Grenada OR Guadeloupe OR Haiti OR Jamaica OR Martinique OR "Puerto Rico" OR "Saint Kitts and Nevis" OR "Saint Lucia" OR "Saint Vincent and the Grenadines" OR "Trinidad and Tobago" OR "United States Virgin Islands" OR Belize OR "Costa Rica" OR "El Salvador" OR Guatemala OR Honduras OR Nicaragua OR Mexico OR Panama OR Argentina OR Bolivia OR Brazil OR Chile OR Colombia OR Ecuador OR "French Guiana" OR Guyana OR Paraguay OR Peru OR Suriname OR Uruguay OR Venezuela)	350

**Other methods**	**Search strategy and keywords**	
ProQuest 19/06/23	“health literacy” AND “Latin America”	92
