# Supplementary material for: The low health literacy in Latin America and the Caribbean: a systematic review and meta-analysis
Source: BMC Public Health. 2024 Jun 1;24:1478. doi: 10.1186/s12889-024-18972-2 (PMC11144327; doi:10.1186/s12889-024-18972-2)
Supplement: Supplementary file 2 — Supplementary Material 2 [file 12889_2024_18972_MOESM2_ESM.pdf]

Additional file 2. List of excluded studies.

No prevalence data (n=31)

1. Bernardes JR, Mattoso CL de Q, Bouzada MAC, Araujo CAS. Vulnerability of poorly literate adult consumers regarding over-the-counter drugs. IJPHM. 2021;15(2):212–234.

2. Cruvinel AFP, Méndez DAC, Oliveira JG, et al. The Brazilian version of the 20-item rapid estimate of adult literacy in medicine and dentistry. PeerJ. 2017;5e3744.

3. Fletcher HF. The Association of Health Literacy with Self-care Agency in Older Adults in Jamaica. Loma Linda University Electronic Theses, Dissertations & Projects.; 2014.

4. Lemley SM, Castro-Díaz S, Cubillos L, et al. Calidad de vida relacionada a salud y alfabetización en salud en pacientes adultos en centros de atención primaria con afiliación al régimen subsidiado o contributivo en Colombia. Revista Colombiana de Psiquiatría. 2021;5023–31.

5. Rivero-Méndez M, Suárez E, Hernández G, et al. Internal Consistency of the Spanish Health Literacy Test (TOFHLA-SPR) for Puerto Rico. PRHSJ. 2010;29(1):6.

6. Taylor PC, Ancuta C, Nagy O, et al. Treatment Satisfaction, Patient Preferences, and the Impact of Suboptimal Disease Control in a Large International Rheumatoid Arthritis Cohort: SENSE Study. PPA. 2021;15359–373.

7. Al-Rousan T, Pesantes MA, Dadabhai S, et al. Patients’ perceptions of self-management of high blood pressure in three low- and middle-income countries: findings from the BPMONITOR study. Glob Health Epidemiol. 2020;5e4.

8. Dongarwar D, Salihu HM. Influence of Sexual and Reproductive Health Literacy on Single and Recurrent Adolescent Pregnancy in Latin America. Journal of Pediatric and Adolescent Gynecology. 2019;32(5):506–513.

9. Evans-Lacko S, Gronholm P, Ribeiro W, Laurens K, Fusar-poli P. Levels of and implications for personal stigma and mental health literacy in relation to psychosis among young people with and without risk of developing psychotic disorder. Concurrent Symposia;Abstracts for the Sixth Biennial SIRS Conference

10. Fernandes DE, Riguetti MTP, Kirsztajn GM. Face Mask Use During the COVID-19 Outbreak: How Did Educated Brazilians Behave? Am J Health Promot. 2021;35(7):991–996.

11. Galo J, Feeney M, Zambrano K, Galo C, Clinchot D. Comprehensive evaluation of male health in four communities in rural Honduras. Preventive Medicine Reports. 2018;1246–53.

12. Maloney CA, Abel WD, McLeod HJ. Jamaican adolescents' receptiveness to digital mental health services: A cross-sectional survey from rural and urban communities. Internet Interventions. 2020;21100325.

13. Moraes KL, Brasil VV, Mialhe FL, et al. Validação do Health Literacy Questionnaire (HLQ) para o português brasileiro. Acta Paulista de Enfermagem. 2021;34eAPE02171.

14. Moura N dos S, Lopes BB, Teixeira JJD, Oriá MOB, Vieira NFC, Guedes MVC. Literacy in health and self-care in people with type 2 diabetes mellitus. Rev Bras Enferm. 2019;72(3):700–706.

15. Gomes Ramalho de Oliveira J, Askari M, Cabral Dias D, et al. Use of novel e-health technologies and its acceptability by kidney transplanted patients: first experience in northeast Brazil. Nephrology Dialysis Transplantation. 2020;35(Supplement_3):1862.

16. Quimby-Worrell C. Health Literacy and Type 2 Diabetes in Barbados. The Qualitative Report,. 2019;24(2):319–332.

17. Russo M. Estudio exploratorio del impacto del alfabetismo funcional sobre conductas sanitarias deficientes a nivel poblacional. Rev méd Chile. 2015;143(7):856–863.

18. Youssef FF, Bachew R, Bodie D, Leach R, Morris K, Sherma G. Knowledge and attitudes towards mental illness among college students: Insights into the wider English-speaking Caribbean population. Int J Soc Psychiatry. 2014;60(1):47–54.

19. Cardoso MCLR, Santos ASF, Fonseca ADG, Silva RF da, Carvalho PD de, Martins AME de BL. Validity and reliability of the Health Literacy Assessment Scale for adherence to drug treatment among diabetics. Einstein (São Paulo). 2019;17(2):eAO4405.

20. Monsalves MJ, Mañalich J, Fuentes E. Validación del test Short Assessment of Health Literacy for Spanish-speaking Adults en Chile, para medir alfabetización en salud. Rev méd Chile. 2016;144(5):604–610.

21. Paskulin LMG, Aires M, Valer DB, Morais EP de, Freitas IB de A. Adaptaçâo de um instrumento que avalia alfabetização em saúde das pessoas idosas. Acta paul enferm. 2011;24(2):271–277.

22. Quemelo PRV, Milani D, Bento VF, Vieira ER, Zaia JE. Literacia em saúde: tradução e validação de instrumento para pesquisa em promoção da saúde no Brasil. Cad Saúde Pública. 2017;33(2)

23. Saavedra-Dahm O, Solar P, Díaz H, et al. La Heterogeneidad del Alfabetismo en Salud y el Consentimiento Informado en Chile. Ter Psicol. 2012;30(3):127–131.

24. Escobar AM, Codner E, Giraudo F, Libman I, Charron-Prochownik D. Using a culturally and linguistically competent health promotion approach to adapt and validate the preconception counseling READY-girls program for Spanish-speaking teens with diabetesIn: Late Breaking Abstracts. Boston, USA 2019:242.

25. Holder-Nevins D, Williams S, Miller Z, Chin D, Grant A, Webster-Kerr K. Issues of health literacy in making sense of the mosquito-chikungunya-fever connection: A Jamaican experienceIn: 25th Annual Research Conference and Workshop on One Health, One Caribbean. 2016:24.

26. Bray L, Carter B, Blake L, Saron H, Kirton JA, Robichaud F, et al. (2021)"People play it down and tell me it can't kill people, but I know people are dying each day". Children's health literacy relating to a global pandemic (COVID-19); an international cross sectional study. PLoS ONE 16(2): e0246405. https://doi.org/10.1371/journal. pone.0246405

27. Jacinto AF, Hill K, Toye C, Ferreira M, Bertoni A, Slatyer S, et al.. Test-retest reliability of the Health Literacy Questionnaire (HLQ-Br) in Brazilian carers of older people. Rev Assoc Med Bras [Internet]. 2021Apr;67(4):500–4. Available from: https://doi.org/10.1590/1806-9282.20201102

28. Oliveira GKA, Moraes KL, Caetano TA, Santos DCG, Oliveira TMM, Borges CJ. Perfil de letramento em saúde de portadores de doença renal crônica em tratamento pré-dialítico. J. nurs. health. 2022;12(1):e2212121016. Disponível em: https://periodicos.ufpel.edu.br/ojs2/index.php/enfermagem/article/view/21016

29. Pimentel SM, Avila MAG, Prata RA, Nunes HRC, Silva JB. Association of health literacy, COVID-19 threat, and vaccination intention among Brazilian adolescents. Rev. Latino-Am. Enfermagem. 2022;30(spe):e3759; Available in: URL: https://doi.org/10.1590/1518-8345.6154.3759

30. Sarkis, L.B.d.S.; Teruel-Camargo, J.; Gibbs, H.D.; Nakano, E.Y.; Ginani, V.C.; de Aguiar, A.S.; Chaves, C.d.S.; Zandonadi, R.P.; Bastos, M.G. The

Nutrition Literacy Assessment Instrument for Brazilians, NLit-Br: An Exploratory Cross-Cultural Validity Study. Nutrients 2022, 14, 4914. https://doi.org/10.3390/nu14224914.

31. Soares TA, Brasil VV, Moraes KL, Santos LT, Vila VS, Borges Júnior LH. Letramento em saúde de cuidadores domiciliares de uma capital brasileira. Acta Paul Enferm. 2021;34:eAPE002255.

Low health literacy as an inclusion criterion (n = 3)

1. de Oliveira SMS, Guimarães DB, Reis JS. Illiteracy and diabetes: educational program for people with type 2 diabetes in the public health system. Diabetol Metab Syndr. 2015;7(S1):A177, 1758-5996-7-S1-A177.

2. Wills J, Sykes S, Hardy S, Kelly M, Moorley C, Ocho O. Gender and health literacy: men's health beliefs and behaviour in Trinidad. Health Promotion International. 2020;35(4):804–811.

3. Trompeter N, Johnco C, Zepeda-Burgos RM, et al. Mental Health Literacy and Stigma Among Salvadorian Youth: Anxiety, Depression and Obsessive-Compulsive Related Disorders. Child Psychiatry Hum Dev. 2022;53(1):48–60.

Study design (non-observational) (n = 10)

1. Doi-Kanno M, Kanoya Y, Moriguchi EH. The effects of a leaflet-based health guide on health literacy, self-efficacy, and satisfaction among older Japanese-Brazilian adults living in Brazil: A quasi-experimental study. BMC Public Health. 2021;21(1):10.

2. Ghisi GL de M, Grace SL, Anchique CV, et al. Translation and evaluation of a comprehensive educational program for cardiac rehabilitation patients in Latin America: A multi-national, longitudinal study. Patient Education and Counseling. 2021;104(5):1140–1148.

3. Lotto M, Strieder AP, Ayala Aguirre PE, et al. Parental-oriented educational mobile messages to aid in the control of early childhood caries in low socioeconomic children: A randomized controlled trial. Journal of Dentistry. 2020;101103456.

4. Serbim A, Paskulin L, Nutbeam D. Improving health literacy among older people through primary health care units in Brazil: feasibility study. Health Promotion International. 2020;35(6):1256–1266.

5. Stonbraker S, Haight E, Lopez A, et al. Digital Educational Support Groups Administered through WhatsApp Messenger Improve Health-Related Knowledge and Health Behaviors of New Adolescent Mothers in the Dominican Republic: A Multi-Method Study. Informatics. 2020;7(4):51.

6. de Almeida JMC, Serra EB, Pascoal LM, Neto MS, Moreira RP, Palmeira Rolim ILT. Analysis of Readiness for enhanced health literacy in patients with

type 2 diabetes mellitus [published online ahead of print, 2023 Mar 31]. Int J Nurs Knowl. 2023;10.1111/2047-3095.12416. doi:10.1111/2047-3095.12416

7. Küchler, Mahara Louíse; Mantovani, Maria de Fátima; Paes, Robson Giovani; Paz, Vanêssa Piccinin; Gribner, Fernanda Cegan; Silva, Emanuele Cristina de Sousa.Intervenções educativas remotas para o letramento de adultos com hipertensão arterial na atenção primária. Ciênc. cuid. saúde ; 21: e61813, 2022

8. Glasinovic Andrés, Rodríguez Claudia, Martín Pamela San, González Diego, Guzmán Rodrigo, Ureta María Del Pilar et al . Efectividad a mediano plazo de un programa multidimensional en personas mayores en centros diurnos en Chile. Rev. méd. Chile [Internet]. 2022 Ene ; 150(1): 23-32.

9. Paes, Robson Giovani. A influência da literacia em saúde e do conhecimento da doença na autogestão do cuidado em adultos com diabetes mellitus tipo 2: subsídios para enfermagem. [recurso eletrônico]: subsídios para a enfermagem - Curitiba, 2021.

10. de Sousa AR, Moreira WC, da Silva Santana T, et al. SARS-CoV-2 in Brazil and Psychosocial Repercussions on Men's Health: Health Literacy Is Important. Am J Mens Health. 2022;16(5):15579883221119091. doi:10.1177/15579883221119091

Studies outside Latin America or the Caribbean (n = 45)

1. Alonso RP, Álvarez PM, García SM, et al. Valoración del nivel de alfabetización en salud en pacientes con insuficiencia renal crónica en tratamiento con hemodiálisis. Enfermería Nefrológica. 2017;20(1):60.

2. Pelayo Alonso R, Martínez Álvarez P, Sánchez Cano MS, Merino García S, Labrador Pérez A, Cobo Sánchez JL. Análisis del nivel de alfabetización en salud, en pacientes con insuficiencia renal crónica en hemodiálisis. Enferm Nefrol. 2017;20(3):221–226.

3. Bacon O, VandenBerg A, May ME. Provider and patient perception of psychiatry patient health literacy. Pharm Pract (Granada). 2017;15(2):908–908.

4. Berry DL, Halpenny B, Chang P, et al. Health literacy screening prior to education for patients with cancer. JCO. 2013;31(31_suppl):155–155.

5. Boyas JF. Correlates of Health Literacy among Latinos in Arkansas. Social Work in Public Health. 2013;28(1):32–43.

6. Castañeda SF, Giacinto RE, Medeiros EA, et al. Academic-Community Partnership to Develop a Patient-Centered Breast Cancer Risk Reduction Program for Latina Primary Care Patients. J Racial and Ethnic Health Disparities. 2016;3(2):189–199.

7. Coffman MJ, Shobe MA, Dmochowski J, Fox SD. Health Care Access and Utilization Among Latino Immigrants. Hisp Hlth Care Int. 2007;5(2):73–80.

8. Conklin JR, Togami JC, Burnett A, Dodd MA, Ray GM. Care Transitions Service: A pharmacy-driven program for medication reconciliation through the continuum of care. American Journal of Health-System Pharmacy. 2014;71(10):802–810.

9. Dawkins-Moultin L, McKyer EL, McDonald A. Abstract C10: Raising HPV awareness in immigrant populations: Are health educators prepared to address health literacy challenges? Cancer Epidemiology, Biomarkers & Prevention. 2018;27(7_Supplement):C10–C10.

10. Guerra CE, Krumholz M, Shea JA. Literacy and Knowledge, Attitudes and Behavior About Mammography in Latinas. Journal of Health Care for the Poor and Underserved. 2005;16(1):152–166.

11. Gunn CM, Paasche-Orlow MK, Bak S, et al. Health Literacy, Language, and Cancer-Related Needs in the First 6 Months After a Breast Cancer Diagnosis. JCO Oncology Practice. 2020;16(8):e741–e750.

12. Hall E, Lee S-Y, Clark PC, Perilla J. Social Ecology of Adherence to Hypertension Treatment in Latino Migrant and Seasonal Farmworkers. J Transcult Nurs. 2016;27(1):33–41.

13. Hashimoto H, Yanagisawa S. Development of health literacy scale among Brazilian mothers in Japan. Health Promot Int. 2016;321034–1040.

14. Hernandez-Mekonnen R, Duggan EK, Oliveros-Rosen L, et al. Health Literacy in Unauthorized Mexican Immigrant Mothers and Risk of Developmental Delay in their Children. J Immigrant Minority Health. 2016;18(5):1228–1231.

15. Hoffman S, Rueda HA, Beasley L. Health-Related Quality of Life and Health Literacy among Mexican American and Black American Youth in a Southern Border State. Social Work in Public Health. 2020;35(3):114–124.

16. Jacobs EA, Walker CM, Miller T, et al. Development and Validation of the Spanish Numeracy Understanding in Medicine Instrument. J GEN INTERN MED. 2016;31(11):1345–1352.

17. King SR, McCaffrey III DJ, Bouldin AS. Health literacy in the pharmacy setting: defining pharmacotherapy literacy. Pharmacy Practice (Internet). 2011;9(4):213–220.

18. Ko N, Festa K, Gunn C, et al. Abstract P3-10-09: Predictors of social support among newly diagnosed breast cancer patients seeking care at an urban safety net academic medical center. Cancer Research. 2017;77(4_Supplement):P3-10–09.

19. Lee-Barber I, Hann G, Singh G, Palit V, Al-Jaddir G. The associations between social factors and suboptimal use of paediatric dental services (Tiny Teeth): a mixed method study. The Lancet. 2019;394S66.

20. Lopez V, Sanchez K, Killian MO, Eghaneyan BH. Depression screening and education: an examination of mental health literacy and stigma in a sample of Hispanic women. BMC Public Health. 2018;18(1):646.

21. Lor M, Koleck TA, Bakken S, Yoon S, Dunn Navarra A-M. Association Between Health Literacy and Medication Adherence Among Hispanics with Hypertension. J Racial and Ethnic Health Disparities. 2019;6(3):517–524.

22. Loureiro L. Estigma pessoal e percebido acerca do abuso de álcool e intenção de procura de ajuda. Rev Enf Ref. 2013;III Série(11):59–66.

23. Lubetkin EI, Zabor EC, Isaac K, Brennessel D, Kemeny MM, Hay JL. Health Literacy, Information Seeking, and Trust in Information in Haitians. Am J Hlth Behav. 2015;39(3):441–450.

24. MacDonald T, MacDonald D, Crooks B, Collicott C. What do children with cancer know about their medications? Pharmacy Practice (Internet). 2011;9(4):207–212.

25. Martins A, Andrade I. Adaptação cultural e validação da versão portuguesa de Newest Vital Sign. Rev Enf Ref. 2014;IV Série(3):75–83.

26. Soto Mas F, Mein E, Fuentes B, Thatcher B, Balcázar H. Integrating Health Literacy and ESL: An Interdisciplinary Curriculum for Hispanic Immigrants. Health Promotion Practice. 2013;14(2):263–273.

27. Soto Mas F, Cordova C, Murrietta A, Jacobson HE, Ronquillo F, Helitzer D. A Multisite Community-Based Health Literacy Intervention for Spanish Speakers. J Community Health. 2015;40(3):431–438.

28. Soto Mas F, Jacobson HE, Olivárez A. Adult Education and the Health Literacy of Hispanic Immigrants in the United States. Journal of Latinos and Education. 2017;16(4):314–322.

29. McDougall JA, Banegas MP, Wiggins C, et al. Disparities in treatment-related financial burden and recurrence in a diverse sample of colorectal cancer survivors. JCO. 2017;35(15_suppl):e18067–e18067.

30. McDougall JA, Banegas MP, Wiggins C, et al. Abstract C52: Disparities in treatment-related financial hardship and adherence to surveillance colonoscopy guidelines in ethnically, linguistically, and geographically diverse colorectal cancer survivors. Cancer Epidemiology, Biomarkers & Prevention. 2018;27(7_Supplement):C52–C52.

31. Moore E, Cordero C. Understanding the Gap: Perceived Health Literacy Levels Among Spanish-Speaking Immigrants in Miami-Dade County, 2016. J Immigrant Minority Health. 2019;21(1):204–209.

32. Al Omar M, Hasan S, Palaian S, Mahameed S. The impact of a self-management educational program coordinated through WhatsApp on diabetes control. Pharm Pract (Granada). 2020;18(2):1841.

33. Paiva D, Silva S, Severo M, Moura-Ferreira P, Lunet N, Azevedo A. Validation of the Short Assessment of Health Literacy in Portuguese-speaking Adults in Portugal. Gaceta Sanitaria. 2020;34(5):435–441.

34. Tomás C, Queirós P, Ferreira T. Análise das propriedades psicométricas da versão portuguesa de um instrumento de avaliação de e-Literacia em Saúde. Rev Enf Ref. 2014;IV(2):19–28.

35. van Servellen G, Brown JS, Lombardi E, Herrera G. Health Literacy in Low-Income Latino Men and Women Receiving Antiretroviral Therapy in Community-Based Treatment Centers. AIDS Patient Care and STDs. 2003;17(6):283–298.

36. 395 Social Determinants Differ Between US and Caribbean/Central American Born Patients with CKD/ESKD: Nutritional Literacy, Diet Quality and Social Support. American Journal of Kidney Diseases. 2019;73(5):746–747.

37. Wolpin SE, Nguyen JK, Parks JJ, et al. Redesigning pictographs for patients with low health literacy and establishing preliminary steps for delivery via smart phones. Pharm Pract (Granada). 2016;14(2):686–686.

38. Ghaddar S, Byun J, Krishnaswami J. Health insurance literacy and awareness of the Affordable Care Act in a vulnerable Hispanic population. Patient Education and Counseling. 2018;101(12):2233–2240.

39. Ghaddar S, Vatcheva KP, Alvarado SG, Mykyta L. Understanding the Intention to Use Telehealth Services in Underserved Hispanic Border Communities: Cross-Sectional Study. J Med Internet Res. 2020;22(9):e21012.

40. Penaranda E, Diaz M, Noriega O, Shokar N. Evaluation of Health Literacy among Spanish-Speaking Primary Care Patients Along the US–Mexico Border: Southern Medical Journal. 2012;105(7):334–338.

41. Diggs A, Ferrigno M, Wei Liu X, et al. Relationship between healthliteracy, education, illnessperception and PCP follow upin inner-city CKD patientsIn: National Kidney Foundation 2018 Spring Clinical Meeting. 2018.

42. Leung L. Intensive, multidisciplinary care management program toimprove diabetes control in an outpatient general medicalpracticeIn: 41st Annual Meeting of the Society of General Internal Medicine, SGIM 2018. 2018:811–812.

43. Perel-Winkler A, Neville A, Nguyen S, et al. Low Health Literacy Does Not Impact Adherence toHydroxychloroquine in Patients with Systemic Lupus. Arthritis Rheumatol. 2017;69.

44. Rojas-Guyler L, Britigan DH, Murnan J, King K, Vaughn LM. Measuring English Linguistic Proficiency and Functional Health Literacy Levels in Two Languages: Implications for Reaching Latino Immigrants. Health Educator. 2013;45(2):2–11.

45. Williams ED, Whitaker KL, Piano M, Marlow LAV. Ethnic differences in barriers to symptomatic presentation in primary care: A survey of women in England. Psycho-Oncology. 2019;28(12):2336–2343.

University students or health professionals (n = 13)

1. Des Courtis N, Lauber C, Costa CT, Cattapan-Ludewig K. Beliefs about the mentally ill: A comparative study between healthcare professionals in Brazil and in Switzerland. International Review of Psychiatry. 2008;20(6):503–509.

2. Fresán A, Berlanga C, Robles-García R, Álvarez-Icaza D, Vargas-Huicochea I. Alfabetización en salud mental en el trastorno bipolar: Asociación con la percepción de agresividad y el género en estudiantes de medicina. Salud Ment. 2013;36(3):229.

3. Mogobe KD, Shaibu S, Matshediso E, et al. Language and Culture in Health Literacy for People Living with HIV: Perspectives of Health Care Providers and Professional Care Team Members. AIDS Research and Treatment. 2016;20161–10.

4. Silva VM, Brasil VV, Moraes KL, Magalhães JPR. Letramento em saúde dos profissionais de um Programa de Residência Multiprofissional em Saúde. Rev Eletr Enferm. 2020;22(62315):1–9.

5. Wildman A. Predictors of Intention to Use Health Literacy Strategies Among Physicians in Grenada. Health Psychology. 1998;147.

6. Instrumento de medición de la alfabetización en salud sexual y reproductiva en estudiantes universitarios. 2018;2211.

7. Logullo P, Torloni MR, de O. C. Latorraca C, Riera R. The Brazilian Portuguese Version of the DISCERN Instrument: Translation Procedures and Psychometric Properties. Value in Health Regional Issues. 2019;20172–179.

8. Valdivieso Mora E. Mental Health Literacy toward Schizophrenia in College Students from El Salvador and the United States of America. Univ Psychol. 2017;16(1)

9. Noblin A, Gabriel MH, Cortelyou-Ward K, Holmes K. Health literacy among visiting college students in the U.S.: A pilot study. Journal of American College Health. 2022;70(2):589–597.

10. Hall KE, Miller MA, Fialkowski VA, et al. Assessment of a community health worker training program in the Peruvian Amazon demonstrates effective learning retention: Implications for health care education in a resource-limited settingIn: Annals of Global Health. 2014:163.

11. Martins R, Saboga-Nunes L, Farinelli MR, Carascosa MG, Ribeiro P. From health care hospitals to health literate organizations: the case of health literacy of health workers in BrazilIn: Parallel Programme. 2019:183.

12. Orellana AF, Garcia RR, Lopez NM, Vargas-Huicochea I, Cisneros CB. Literacy among medical students on schizophrenia. Salud(i)Ciencia. 2012;19(3):220–223.

13. Mávita-Corral CJ. Alfabetización en salud de una comunidad universitaria del noroeste de México en el año 2016. Inv Ed Med. 2018;7(25):36–45.

Knowledge assessment (n = 8)

1. Casas RN, Gonzales E, Aldana-Aragón E, et al. Toward the early recognition of psychosis among Spanish-speaking adults on both sides of the U.S.–Mexico border. Psychological Services. 2014;11(4):460–469.

2. Collins JH, Bowie D, Shannon G. A descriptive analysis of health practices, barriers to healthcare and the unmet need for cervical cancer screening in the Lower Napo River region of the Peruvian Amazon. Womens Health (Lond Engl). 2019;15174550651989096.

3. Díaz-Correa LM, Ramírez-García LM, Castro-Santana LE, Vilá LM. Osteoporosis knowledge in patients with a first fragility fracture in Puerto Rico.

4. Nachega JB, Morroni C, Zuniga JM, et al. HIV Treatment Adherence, Patient Health Literacy, and Health Care Provider–Patient Communication: Results from the 2010 AIDS Treatment for Life International Survey. Journal of the International Association of Physicians in AIDS Care. 2012;11(2):128–133.

5. Ohnishi M, Nakamura K, Takano T. Improvement in maternal health literacy among pregnant women who did not complete compulsory education: policy implications for community care services. Health Policy. 2005;72(2):157–164.

6. Ravindran AV, Herrera A, Kutcher S, et al. Improving youth well-being in Nicaragua through mental health education: A pilot project. Annals of Global Health. 2016;82(3):597.

7. Pitton Rissardo J, Fornari Caprara AL, Cervi Prado AL. Stroke Literacy in a South Brazilian City: A Community Based Survey. Journal of Stroke and Cerebrovascular Diseases. 2018;27(9):2513–2518.

8. Silva-Tinoco R, Meza D, Martinez D, et al. Diabetes self-management education program effect on glycemic control and insulin dosing in poor diabetes literacy patients from urban areas in Mexico City-therapeutic education

underuse and insulin overuseIn: 78th Scientific Sessions of the American Diabetes Association, ADA 2018. Diabetes; 2018.

Duplicate population (n=6)

1. Apolinario D, Mansur LL, Carthery-Goulart MT, Brucki SMD, Nitrini R. Detecting limited health literacy in Brazil: development of a multidimensional screening tool. Health Promotion International. 2014;29(1):5–14.

2. Souza JG, Apolinario D, Farfel JM, et al. Aplicabilidade do Spoken Knowledge in Low Literacy Patients with Diabetes em idosos brasileiros. Einstein (São Paulo);14(4):7.

3. Cajita MI, Denhaerynck K, Dobbels F, Berben L, Russell CL, De Geest S. Adequate Health Literacy Is Associated with Sufficient Physical Activity: Findings from the BRIGHT StudyIn: The Journal of Heart and Lung Transplantation. 2016.

4. Tenani CF, Silva Junior MF, Sousa M da LR de, Batista MJ. Health literacy dimensions among public health service users with chronic diseases in Piracicaba, Brazil, 2019. Braz J Oral Sci [Internet]. 2022;21:e227259. Available from: https://doi.org/10.20396/bjos.v21i00.8667259

5. Puello SDCP, Silva-Júnior MF, de Sousa MDLR, Batista MJ. Criterion validity of 14-item Health Literacy Scale (HLS-14) questionnaire in Brazilian adults and older people. Health Promot Int. 2022;37(5):daac142. doi:10.1093/heapro/daac142

6. Mialhe FL, Sampaio HAC, Moraes KL, Brasil VV, Rebustini F. Psychometric properties of the Brazilian version of the European Health Literacy Survey Questionnaire short form. Health Promot Int. 2022;37(4):daac130. doi:10.1093/heapro/daac130

Protocols (n=3)

1. Aguirre PEA, Lotto M, Strieder AP, Cruvinel AFP, Cruvinel T. The Effectiveness of Educational Mobile Messages for Assisting in the Prevention of Early Childhood Caries: Protocol for a Randomized Controlled Trial. JMIR Res Protoc. 2019;8(9):e13656.

2. Cartes-Velasquez R, Araya C, Flores R, Luengo L, Castillo F, Bustos A. A motivational interview intervention delivered at home to improve the oral health literacy and reduce the morbidity of Chilean disadvantaged families: a study protocol for a community trial. BMJ Open. 2017;7(7):e011819.

3. Soto F, Roman L, Melin K, Rodriguez A. Impact of clinical pharmacist intervention on medication adherence and health care outcomes among patients with low health literacy in Puerto RicoIn: APhA2015 abstracts of contributed papers. J. Am. Pharm. Assoc.; 2015.

Reviews (n=2)

1. Castillo JL, Palma C, Cabrera-Matta A. Early Childhood Caries in Peru. Front Public Health. 2019;7337.

2. González-Burboa A, Vera-Calzaretta A, Villaseca-Silva P, Müller-Ortiz H. Diabetes Mellitus tipo 2: desafíos para los modelos de cuidados crónicos en Chile. Rev méd Chile. 2019;147(3):361–366.

Abstracts (n=21)

1. Aguirre C, González E, Velázquez P, Macías N, Garnica F, Guzman E. Health literacy level among patients attending a urogynecology practice in West MexicoInt Urogynecol J; 2020:S159.

2. Bernardes C de P, Moraes K, Silva AM, et al. Health literacy among people living with hypertension in rural areasQual Life Res; 2016:125–126.

3. Costa J, Marcolino M, Torres H, et al. The Impact of an Educational Intervention in Patients with Atrial Fibrillation Treated with WarfarinRes Pract Thromb Haemost

4. Japiassú L, Brito G, Castro S, Gomes M. A comparison of the neurobehavioral profile o ftype 1 and type 2 diabetes patientsIn: Diabetes. 2015:Suppl 1.

5. Martins R, Saboga-Nunes L. The challenges of epistemological validation to Brazil of the european health literacy survey (HLS-EU-BR). Atencion Primaria. 2014;4612.

6. Muniz LH, Gomes MB, Negrato CA, Calassara P de C, Dias ALN. Health literacy and glycemic control in patients with diabetes: a tertiary care center study in Brazil. Diabetol Metab Syndr. 2019;11.

7. Oliveira J, Resende R, Sales T, Silva L, Nogueira L. Profile of patients using oral anticoagulants in primary care: 'anticoagula divinópolis'project. Res Pract Thromb Haemost. 2020;4(Suppl 1 (1043))

8. Parra G, Diaz F, Verdejo H, Castro P. Health literacy and heart failure knowledge: identifying the associated factors in chilean population. Eur J Cardiovasc Nurs. 2014;13.

9. Pedraza L, Pokorski S, Machado B, Ferro E, Souza R. Health literacy, knowledge,treatment adhesion and self-care in heart failure in amiddle income country. Eur J Heart Fail. 2019;21 Suppl 1(Suppl 1):5–592.

10. Rey-Ares L, Augustovski F, Irazola V, et al. Health Literacy and Self-Reported Health Status Using the Eq-5d-5l: An Exploratory Analysis. Value in Health. 2014;17(7):A516.

11. Souza e Silva M, Rodrigues L, Oliveira Baldoni A, et al. Implementation of a Text-message Intervention to Warfarin Users in a Brazilian Primary Care Setting. Res Pract Thromb Haemost. 2020;4.

12. Silva-Tinoco R, Meza D, Martinez D, et al. Diabetes self-management education program effect on glycemic control and insulin dosing in poor diabetes literacy patients from urban areas in Mexico City-therapeutic education underuse and insulin overuseIn: 78th Scientific Sessions of the American Diabetes Association, ADA 2018. Diabetes; 2018.

13. Stonbraker S, Castillo H, Lerebours Nadal L, et al. The Health Information Behavior of HIV Positive Adults in La Romana, Dominican Republic. Nurs Res. 2016;65(2)

14. Tenani C, Sousa M, Batista M. Health literacy and associated factors in patients with chronic diseases, Brazil. Eur J Public Health. 2020;30.

15. Verastegui-aviles E, González-Garza S, Allende-Perez D, Campos-Lopez A. Health literacy in mexican cancer patients. Support Care Cancer; 2018:S335.

16. Westlake C, Espinel M, Ortega F. Healthy-heart behaviors, risk factor knowledge, and health-literacy among Ecuador's rural poor. Circulation; 2017.

17. Marcolino M., Oliveira J., Rios D., Pedroso T., Sa L., Parreiras Martins M., Sales T., Ribeiro A. Prevention of stroke and other thromboembolic events in primary care: Alarming data from a Brazilian medium-size city. Research and Practice in Thrombosis and Haemostasis, Supplement 6, 2022.

18. Varsha Muralidhar et al. Differential Quality Of Life Assessments In Haitian And Dominican Patients With Heart Failure: The Scan-mp Study. Journal of Cardiac Failure Vol. 29 No. 4 April 2023.

19. Saboga-Nunes L, Santini LA, da Silveira F, Moro E, Estabel L. Access to health literacy best practices and the role of health libraries (BiblioSUS Network BVS) in Brazil. Eur J Public Health. 2022 Oct 25;32(Suppl 3):ckac129.512. doi: 10.1093/eurpub/ckac129.512. PMCID: PMC9593778.

20. Garcia X., Blanco D.B., Fuentes-Alabi De Aparicio S., Maza M., Benitez S., Mcneil M., Baker J.Series on quality of life for children with cancer: improving palliative care health literacy among parents and caregivers pediatric blood and cancer (s43) supplement 5, 2022.

21. Ishmael, L., Cardet, J. C., Yawn, B., Casale, T., Celedon, J., Busse, P., ... & Israel, E. (2023). Asthma Morbidity Measures Across Black Ethnic Subgroups. Journal of Allergy and Clinical Immunology, 151(2), AB123.

Case report (n=1)

1. Santoso LF, Erkkinen EE, Deb A, Adon C. HIV-associated dementia in the Dominican Republic: a consequence of stigma, domestic abuse and limited health literacy. BMJ Case Reports. 2016;bcr2016214615.

Book (n=1)

1. Atwell-Scrivner J. Building Healthy Bridges: Informal Leadership and Health Literacy. Gonzaga University, Spokane, Wash.; 2010.

Pilot study (n=3)

1. Aguayo L, Sandberg E, Schwingel A, Wiley A. Low Health Literacy Hinders Accurate Perceptions of Body Weight among Mexican Women. FASEB J;31(S1)

2. Wilson FL, Mayeta-Peart A, Parada-Webster L, Nordstrom C. Using the Teach-Back Method to Increase Maternal Immunization Literacy Among Low-Income Pregnant Women in Jamaica: A Pilot Study. Journal of Pediatric Nursing. 2012;27(5):451–459.

3. Janevic MR, Aruquipa Yujra AC, Marinec N, et al. Feasibility of an interactive voice response system for monitoring depressive symptoms in a lower-middle income Latin American country. Int J Ment Health Syst. 2016;10(1):59.

List of excluded studies identified via other methods:

Knowledge assessment (n = 1)

1. Roncada C, Cardoso T de A, Bugança BM, Bischoff LC, Soldera K, Pitrez PM. Levels of knowledge about asthma of parents of asthmatic children. einstein (São Paulo) [Internet]. 2018;16(2):eAO4204. Available from: https://doi.org/10.1590/S1679-45082018AO4204

University students or health professionals (n = 1)

1. Silva VM, Brasil VV, Moraes KL, Magalhães JPR. Health literacy of professionals enrolled in a Multiprofessional Residency Program in Health. Rev. Eletr. Enferm. [Internet]. 2020 ;22:62315. Available at: https://doi.org/10.5216/ree.v22.62315

Duplicate population (n=1)

1. Lima JP de, Abreu DPG, Bandeira E de O, Brum AN, Garlet BB, Martins NFF. Functional health literacy in older adults with hypertension in the Family Health Strategy. Rev Bras Enferm [Internet]. 2020;73:e20190848. Available from: https://doi.org/10.1590/0034-7167-2019-0848

No prevalence data (n=3)

1. Brucki, S. M. D., Mansur, L. L., Carthery-Goulart, M. T., & Nitrini, R.. (2011). Formal education, health literacy and Mini-Mental State Examination. Dementia & Neuropsychologia, 5(1), 26–30. https://doi.org/10.1590/S1980-57642011DN05010005

2. Leão NC de S, Canhestro MR, Milagres LMR, Oliveira PM, Moraes KL, Brasil VV. Health literacy and pharmacotherapy adherence among chronic kidney disease patients in pre-dialysis care / Letramento em saúde e adesão a medicação de doentes renais crônicos em tratamento pré-dialitico. Rev. Pesqui. (Univ. Fed. Estado Rio J., Online) [Internet]. 28º de setembro de 2021 [citado 18º de novembro de 2023];13:1610-7. Disponível em: https://seer.unirio.br/cuidadofundamental/article/view/10792

3. Moraes KL, Brasil VV, Mialhe FL, Sampaio HA de C, Sousa ALL, Canhestro MR, et al.. Validação do Health Literacy Questionnaire (HLQ) para o português brasileiro. Acta paul enferm [Internet]. 2021;34:eAPE02171. Available from: https://doi.org/10.37689/acta-ape/2021AO02171

Pilot study (n=1)

1. de Oliveira MO, Porto CS, Brucki SMD. S-TOFHLA in mild Alzheimer's disease and Mild Cognitive Impairment patients as a measure of functional literacy: Preliminary study. Dement Neuropsychol. 2009 Oct-Dec;3(4):291-298. doi: 10.1590/S1980-57642009DN30400005. PMID: 29213642; PMCID: PMC5619414.
