# Supplementary material for: The low health literacy in Latin America and the Caribbean: a systematic review and meta-analysis
Source: BMC Public Health. 2024 Jun 1;24:1478. doi: 10.1186/s12889-024-18972-2 (PMC11144327; doi:10.1186/s12889-024-18972-2)
Supplement: Supplementary file 3 — Supplementary Material 3 [file 12889_2024_18972_MOESM3_ESM.pdf]

Additional file 3. List of individual characteristics of included studies.

Study	Country	Total	Age (median, mean or categorized)	Education	Tools	Sex	High HL	Intermediate HL	Low HL	Idiom	Tools validation	Setting	Target population
**Abbott et al., 2018**	Bolivia	643	53 ± 15	<9 years: 269<br>9-11: 177<br>12 or more: 187	a single question based on the work of Wallace, Rogers, Roskos, Holliday, & Weiss (2006)	Male+female: 651*<br>Male: 241<br>Female: 410		271	372	Spanish	Not validated in the country of application	Health services and others	Other pathologies
**Aguayo-Verdugo et al., 2019**	Chile	175	65.1 ± 11.0	9,3 ± 4,2	SAHLSA-50	Male+female: 175<br>Male: 52<br>Female: 123	140		35	Spanish	Validated in the country	Health services and others	Diabetic patients
**Almeida et al., 2019**	Brazil	80	54.6±11.7	1-4 years: 19<br>5-8 years: 15<br>9-11 years: 17<br>≥12 years: 29	S-TOFHLA	Male+female: 80<br>Male: 10<br>Female: 70	58	10	12	Portuguese	Validated in the country	Health services and others	Caregivers and parents
**Apolinario et al., 2012**	Brazil	226	74.4 ± 6.9	5.3 ± 4.0	SAHLPA-50	Male+female: 226<br>Male: 64<br>Female: 162	77		149	Portuguese	Validation study	Health services and others	Older people
**Apolinario et al., 2015**	Brazil	322	47.2 ± 16.8	9.6 ± 5.2	S-TOFHLA	Male+female: 322<br>Male:11	220	28	74	Portuguese	Validated in the country	Health services and others	Health service users

						8<br>Female: 204							
**Bartolazzi et al., 2021**	Brazil	100	68.8±13.8	Elementary school 74<br>High school 20<br>Beyond high school 6	SAHLPA-18	Male+female: 100<br>Male: 46<br>Female: 54	21		79	Portuguese	Validated in the country	Health services and others	Heart disease patients
**Batista et al., 2020**	Brazil	143	23 - 48 years: 68<br>49 - 69 years: 75	> 11 years: 78<br>5 - 10 years: 34<br>< 4 years: 31	HLS-14	Male+female: 143<br>Male: 103<br>Female: 40	77		66	Portuguese	Validation study	Households	General population
**Bezerra et al., 2019**	Brazil	42	23 - 40 years:17<br>41 - 59 years: 15<br>> 60 years: 10	< 4 years: 2<br>4 - 7 years: 10<br>8 - 10 years: 10<br>> 11 years: 20	S-TOFHLA	Male+female: 42<br>Male: 16<br>Female: 26	8		34	Portuguese	Validated in the country	Health services and others	Patients with nephropathies
**Bhawra et al., 2023**	Mexico	4044	18–29 years:1204<br>30–44 years:1305<br>45–59 years:1155<br>60+ years:380	Low 789<br>Medium 535<br>High 2720	adapted version of the NVS	Male: 1925<br>Female: 2119	1549	1261	1234	Spanish	Not validated	web-based surveys	General population
**Bolivar et al., 2017**	Argentina	156	62.6 ± 12.3	≤ 7 years: 79<br>8 - 12 years: 61<br>> 12 years:	SAHLSA-50	Male+female: 156<br>Male: 65	62		94	Spanish	Validated in language but not country	Health services and others	Diabetic patients

				14<br>Missing 2 (		Female:<br>91							
**Borges et al., 2019**	Brazil	357	18-39 years:158<br>40-60 years: 146<br>>60 years:53	Incomplete elementary school 119 (33,8%)<br>Elementary school 65 (18,4%)<br>High school 135 (38,2%)<br>Higher education 34 (9,6%)	S-TOFHLA	Male+female: 357<br>Male: 97<br>Female: 260	126	45	18<br>6	Portuguese	Validated in the country	Health services and others	General population
**Borges et al., 2022**	Brazil	251	20-40 years: 32<br>40-60 years: 219	Incomplete Elementary School 96<br>Complete Elementary School 37<br>Complete High school 90<br>Complete higher education 24	S-TOFHLA	Female: 185<br>Male: 66	45		20<br>6	Portuguese	Validated in the country	Health services and other	Hypertensive patients
**Bourne et al., 2010**	Jamaica	355	438 men were ages 75 years and older; 712 men were ages 64.5 to 74 years and 852 men reported	No formal education 200<br>Primary and infant 1661<br>Secondary 102<br>Tertiary 37	Not reported	Male+female: 355<br>Male: 355<br>Female: 0	184		17<br>1	English	Not validated	Households	General population

			ages 55 to 64 years										
**Cajita et al., 2017**	Brazil	98	45.2 ± 13.2	Primary 25 Secondary 50 University 20	single-item variant of the Subjective Health Literacy Screener (SHLS)	Male+female: 98 Male: 65 Female: 33	36		62	Portuguese	Not validated in the country of application	Health services and others	Heart disease patients
**Campos et al., 2020**	Brazil	439	42.5 ± 10,6	8 years	S-TOFHLA	Male+female: 439 Male: 0 Female: 439	204		235	Portuguese	Validated in the country	Health services and others	General population
**Cangussú et al., 2021**	Brazil	372	≤30 years: 62 31-59 years: 217 ≥60 years: 93	Incomplete Elementary School 146 Complete Elementary School 27 Incomplete High school 29 Complete High school 124 Incomplete higher education 10 Complete higher education 36	S-TOFHLA	Male: 271 Female: 101	142	42	188	Portuguese	Validated in the country	Health services and other	Health service users

**Cardoso et al., 2021**	Brazil	125	35.7±9.2	Elementary school 57<br>High school 65<br>Higher education 3	SAHLPA-18	Male+female: 125<br>Male: 64<br>Female: 61	51		74	Portuguese	Validated in the country	Health services and others	Caregivers and parents
**Carrilho et al., 2022**	Brazil	326	61.22 ± 10.32		SAHLPA-18	Male: 110<br>Female: 216	101		225	Portuguese	Validated in the country	Health services and other	Hypertensive patients
**Carthery-Goulart et al., 2009**	Brazil	312	47.3 ± 16.8	9.9 ± 5	S-TOFHLA	Male+female: 312<br>Male: 114<br>Female: 198	211	28	73	Portuguese	Validation study	Health services and others	Health service users
**Carvalho and Ribeiro, 2020**	Brazil	340	0 - 49 years: 35<br>50 - 89 years: 301	Illiterate: 40<br>Elementary School: 266<br>High school, incomplete e complete higher education: 33	S-TOFHLA	Male+female: 340<br>Male: 107<br>Female: 233	67		273	Portuguese	Not validated	Households	Hypertensive patients
**Cavalcante et al., 2020**	Brazil	286	24 to 59 years:160<br>60 to 92 years: 126	Over 8 years: 106<br>Up to 8 years: 180	Rapid Estimate of Adult Literacy in Medicine (REALM)	Male+female: 286<br>Male: 51<br>Female: 235		129	157	Portuguese	Not validated in the country of application	Health services and others	Hypertensive patients
**Chehuen Neto et al., 2019**	Brazil	345	55 ± 12.5	< 4 years: 153<br>> 4 years: 192	S-TOFHLA	Male+female: 345<br>Male:	170	71	104	Portuguese	Validated in the country	Health services and others	Heart disease patients

						112 Female: 233							
**Coelho et al., 2014**	Brazil	176	44.4 (19-59)	73 had finished high school and 59 had not finished elementary school	S-TOFHLA	Male+female: 176 Male: 43 Female: 133	74	16	86	Portuguese	Validated in the country	Health services and others	Health service users
**Costa et al., 2021**	Brazil	76	± 63 years	Illiterate 12 Incomplete Elementary School 40 Complete Elementary School 13 Incomplete High school 4 Complete High school 6 Complete higher education 1	SAHLPA-18	Male: 48 Female: 28	11		65	Portuguese	Validated in the country	Health services and other	Heart disease patients
**de Castro, 2014**	Brazil	150	≤ 45 years: 21 46 – 60 years:62 ≥ 61 years: 67	Less than 10 years: 74 10 to 12 years:61 College or higher: 15	S-TOFHLA	Male+female: 164* Male: 78 Female: 86	110	17	23	Portuguese	Validated in the country	Health services and others	Diabetic patients
**Diemer et al., 2017**	Suriname	99	44.9 ± 13.4	<8 years: 32 8–12 years: 35 >12 years: 32	Rapid Estimate of Adult Literacy in Medicine in Dutch	Male+female: 99 Male: 51 Female: 48	35	30	34	Dutch	Validated in language but not country	Health services and others	General population

					(REALM-D)								
**Dingemans et al., 2018**	Honduras	22	NR	At least high school education 3	BRIEF Health Literacy Screen	Male+female: 22 Male: 14 Female: 8	3		19	Spanish	Not validated in the country of application	Health services and others	Caregivers and parents
**Doubova et al., 2019**	Mexico	778	62.3 ± 10.9	Without schooling or incomplete elementary school 170 Complete elementary school, with or without secondary school 374 High school or higher 234	HLS-EU-Q47	Male+female: 778 Male: 253 Female: 525	137	462	179	Spanish	Validated in language but not country	Health services and others	Diabetic patients
**Durán et al., 2021**	Chile	733	67(61-73)	Basic 311 Highschool 324 Superior 76	SAHLSA-50	Male+female: 733 Male: 147 Female: 586	611		122	Spanish	Validated in the country	Health services and others	Health service users
**Figueroa Saavedra et al., 2020**	Chile	119	71 (60-88)	NR	SAHLSA-50	Male+female: 119 Male: NR Female: NR	91		28	Spanish	Validated in the country	NR	Older people

**Flower et al., 2016**	Barbados	106	42.6	13.7 years	Rapid Estimate of Adult Literacy in Medicine–Short Form (REALM-SF)	Male+female: 106<br>Male: 0<br>Female: 106	85		21	English	Validated in language but not country	Health services and others	Other pathologies
**França et al., 2020**	Brazil	86	23 (17-25)	NR	SAHLPA-18	Male+female: 86<br>Male: 0<br>Female: 86	23		63	Portuguese	Validated in the country	Health services and others	General population
**Ghisi et al., 2022**	Brazil	159	62.7±10.1	Elementary School or less 57<br>High school 65<br>Higher education 37	BRIEF Health Literacy Screening Tool	Male: 96<br>Female: 62<br>NR: 1	15<br>NR:1	56	87	Portuguese	Not validated in the country of application	web-based surveys	Heart disease patients
**Gordon Singh and Aiken, 2017**	Jamaica	88	18-30 years: 0<br>31-50 years: 17<br>51-70 years: 54<br>>70 years: 17	None 0<br>Primary 16<br>All Age/Junior High 34<br>Secondary 28<br>Technical/Vocational 8<br>College/University 2	NVS	Male+female: 88<br>Male: 20<br>Female: 68	12	52	24	English	Validated in language but not country	Health services and others	Diabetic patients
**Hadden et al., 2018**	Puerto Rico	751	46.1± 11.2	Less than HS 227<br>Graduate equivalency degree 213<br>High school	NVS	Male+female: 751<br>Male: 640	303		448	Spanish	Validated in language but not country	Health services and others	General population

				graduate 121 Some college/college graduate 184 Unknown 6		Female: 111							
**Hagger et al., 2018**	Brazil	100	48.73 ± 15.57	Lower education 62 Higher education 38	Health literacy screening questions (HLSQ)	Male+female: 100 Male: 39 Female: 61	78		22	Portuguese	Not validated in the country of application	Health services and others	Health service users
**Hoffman et al., 2017**	Guatemala	210	12.6 ± 1.01	junior high schools	NVS	Male+female: 210 Male: 115 Female: 95	59	116	35	Spanish	Validated in the country	Schools	Children and adolescents
**Inés and Rosa, 2018**	Peru	200	51.36 ± 16.49	Incomplete elementary school 40 Elementary school 36 Incomplete high school 34 High school 50 Incomplete higher education 18 Higher education 22	SAHLSA-50	Male+female: 200 Male: 66 Female: 134	114		86	Spanish	Validated in language but not country	Health services and others	Other pathologies

**Konfino et al., 2009**	Argentina	229	19-50 years: 95<br>51-64 years: 49<br>65-93 years: 85	< 8 years: 92<br>8-12 years: 85<br>>12 years: 51	SAHLSA-50	Male+female: 229<br>Male: 104<br>Female: 125	160		69	Spanish	Validated in language but not country	Health services and others	Health service users
**León-Landa et al., 2019**	Mexico	90	59.4 ± 11.3	No formal education 27<br>Elementary School 25<br>High school 25<br>Higher education or more 13	HLS-EU-Q47	Male+female: 90<br>Male: 25<br>Female: 65	6	43	41	Spanish	Validated in language but not country	Health services and others	Diabetic patients
**Lima et al., 2019**	Brazil	350	High HL: 65.6± 4.1<br>Intermediate HL: 66.9 ± 5.6<br>Low HL: 69.8 ± 6.4	High HL: 7.4 ± 3.1<br>Intermediate HL: 5.8 ± 2.6<br>Low HL: 3.8 ± 2.1	S-TOFHLA	Male+female: 350<br>Male: 114<br>Female: 236	86	58	206	Portuguese	Validated in the country	Households	Older people
**Lima et al., 2020**	Brazil	60	60-69 years: 36<br>70-79 years: 21<br>>80 years: 3	≤ 4 years: 35<br>>4 years: 25	S-TOFHLA	Male+female: 60<br>Male: 38<br>Female: 22	17		43	Portuguese	Validated in the country	Health services and others	Older people
**Lima et al., 2022**	Brazil	400	33.5±11.85	Incomplete Elementary School 81<br>Complete Elementary School 49<br>Incomplete	S-TOFHLA	Male: 82<br>Female: 318	236	66	98	Portuguese	Validated in the country	Health services and other	Health service users

				High school 49 Complete High school 166 Incomplete higher education 30 Complete higher education 25									
**Macha-Quillama et al., 2017**	Peru	363	43.69 ± 14.43	No formal education 1 Incomplete Elementary School 24 Elementary School 41 Incomplete High school 77 High school 114 Incomplete Higher education 62 Higher education 44	SAHLSA-50	Male+female: 363 Male: 97 Female: 266	238		125	Spanish	Validation study	Health services and others	Health service users
**Manola et al., 2020**	Brazil	30	18 years:7 19-25 years:20 26-40 years:3	Incomplete Elementary School 3 Elementary School 5 Incomplete High school 6 High school	S-TOFHLA	Male+female: 30 Male: 0 Female: 30	23	1	6	Portuguese	Not validated	Health services and others	General population

				12 Incomplete Higher education 3 Higher education 1									
**Maragno et al., 2019**	Brazil	302	46.6	1 - 3 years:58 4 - 7 years:112 8 - 11 years:103 ≥ 12 years: 27	TOFHLA	Male+female: 302 Male: 83 Female: 219	165	58	79	Portuguese	Validation study	Health services and others	Health service users
**Marques and Lemos, 2018**	Brazil	368	37.0 ± 11.0	11.0 ± 3.8	SAHLPA-18	Male+female: 368 Male: 177 Female: 191	94		274	Portuguese	Validated in the country	Health services and others	Health service users
**Martins et al., 2017**	Brazil	422	62.1 (53.9; 71.8)	5.0 (4.0; 8.0)	SAHLPA-18	Male+female: 422 Male: 174 Female: 248	117		305	Portuguese	Validated in the country	Health services and others	Other pathologies
**McNaughton et al., 2015**	Guyana	228	43(interquar-tile range [IQR] 38 to 53)	None 12 Primary 83 Secondary 108 Tertiary 23	Single Item Literacy Screener (SILS)	Male+female: 228 Male: 103 Female: 125	125		103	English	Validated in language but not country	Health services and others	Health service users

**Medina et al., 2022**	Brazil	196	57 ± 8.39	130 had less than 9 years of study	TOFHLA	Male+female: 196 Male: 94 Female: 102	112	46	38	Portuguese	Not validated	Health services and others	Diabetic patients
**Mialhe et al., 2021**	Brazil	783	38.6 ± 14.5	Elementary school 262	European Health Literacy Survey Questionnaire short-short form (HLS-EU-Q6)	Male+female: 783 Male: 250 Female: 533	16	405	362	Portuguese	Validation study	Households	Health service users
**Mora Vicarioli et al., 2021**	Costa Rica	51	34	No formal education 39 Elementary school 8 Incomplete elementary school 4	SAHL-S&E	Male+female: 51 Male: 22 Female: 29	0		51	Spanish	Validated in language but not country	Health services and others	Health service users
**Moraes et al., 2017**	Brazil	60	< 60 anos 25 (41,7) ≥ 60 anos 35 (58,3)	Alfabetizado (< 1 ano de estudo) 17 (28,3) < 9 anos de estudo 26 (43,4) ≥ 9 anos de estudo 17 (28,3)	S-TOFHLA	Male+female: 60 Male: 23 Female: 37	0		60	Portuguese	Validated in the country	Health services and others	Patients with nephropathies
**Oliveira et al., 2014**	Brazil	148	HC: 70.66 MCI: 72.64 AD: 75.80	HC: 8.72 MCI:7.14 AD:6.96	S-TOFHLA	Male+female: 148 Male: 60	52	23	73	Portuguese	Validated in the country	Health services and others	Other pathologies

						Female: 88							
**Oscalices et al., 2019**	Brazil	100	63.3 ±15.2	0 a 4.9 years: 40<br>5 a 8.9 years: 36<br>>9 a 11.9 years: 24	NVS	Male+female: 100<br>Male: 43<br>Female: 57	11	21	68	Portuguese	Validated in the country	Health services and others	Heart disease patients
**Paes et al., 2022**	Brazil	33	57.0±8.08	≤4 years: 6<br>>4 years: 26	Eight-Item Health Literacy Assessment Tool (HLAT-8)	Male: 23<br>Female: 10	19		14	Portuguese	Validated in the country	Health services and other	Diabetic patients
**Pasklan et al., 2021**	Brazil	121	60 to 64 years: 48<br>65 to 69 years: 30<br>70 to 74 years: 19<br>75 to 79 years: 16<br>≥80 years: 8	No formal education 5<br>Elementary School 91<br>High school 20<br>Incomplete higher education 2	S-TOFHLA	Male: 47<br>Female: 74	20	28	73	Portuguese	Not validated	Health services and other	Health service users
**Pavão et al., 2021**	Brazil	107	57.8±14.2	<8 years: 55<br>> 8 years: 52	HLS-EU-BR	Male: 30<br>Female: 77	5	57	40	Portuguese	Validated in the country	Health services and other	Diabetic patients
**Penaloza et al., 2019**	Peru	272	39 (29–52)	None/primary 59<br>Secondary 125<br>Technical 40<br>University 48	SAHL-S&E	Male+female: 272<br>Male: 92<br>Female: 180	193		79	Spanish	Validated in language but not country	Health services and others	General population

**Pereira Cruvinel et al., 2018**	Brazil	250	37.52 ±15.01	8th grade: 59<br>9–12th grade: 90<br>College: 70<br>Postgraduation: 31	NVS	Male+female: 250<br>Male: 90<br>Female: 160	67	58	125	Portuguese	Validation study	Oral health services and others	General population
**Pinhati et al., 2019**	Brazil	485	62 ± 12.6	4.9 ± 3.8	SAHLPA-18	Male+female: 485<br>Male: 212<br>Female: 273	141		344	Portuguese	Validated in the country	Health services and others	Hypertensive patients
**Ribeiro et al., 2021**	Brazil	49	58.4 ± 16.8	Incomplete Elementary School to Incomplete High school 25<br>High school to higher education 24	SAHLPA-18	Male: 16<br>Female: 33	23		26	Portuguese	Validated in the country	Health services and other	Other pathologies
**Rivero-Méndez et al., 2015**	Puerto Rico	199	46.6± 9.54	Less than high school: 68<br>High school: 67<br>More than high school: 65	Test of Functional Health Literacy in Adults-Spanish – Puerto Rico version (TOFHLA-SPR)	Male+female: 200*<br>Male: 98<br>Female: 102	105	43	51	Spanish	Validated in the country	Health services and others	HIV patients
**Rocha and**	Brazil	138	52.0±15.5	Incomplete elementary school 23	SAHLPA-18	Male+female: 138	67		71	Portuguese	Validated in the country	Health services	Patients with

**Figueiredo, 2019**				Elementary school 28<br>Incomplete high school 11<br>High school 59<br>Higher education 17		Male: 80<br>Female: 58						and others	nephropathies
**Rocha et al., 2017**	Brazil	384	17.1 ±15-19	attending high school	10 self-reported questions to assess the functional health literacy	Male+female: 384<br>Male: 114<br>Female: 270	198		186	Portuguese	Validated in the country	Schools	Children and adolescents
**Rocha et al., 2019**	Brazil	78	58.15±11.44	8.81±5.29	S-TOFHLA	Male+female: 78<br>Male: 22<br>Female: 56	25	13	40	Portuguese	Not validated in the country of application	Health services and others	Diabetic patients
**Rodrigues et al., 2017**	Brazil	189	42.1 ± 10	Incomplete primary education 50<br>Complete primary education 85<br>Complete college 34<br>Postgraduate 20	NVS	Male+female: 189<br>Male: 63<br>Female: 126	92	49	48	Portuguese	Validation study	Health services and others	General population

**Rodriguez et al., 2021**	Puerto Rico	1911	18–29 years: 481<br>30–39 years: 361<br>40–49 yeras: 426<br>≥50 years: 643	High school graduate or less 70<br>Associate degree 168<br>Some college 101<br>Undergraduate degree 691<br>Masters 537<br>Doctoral degree 344	asking about the participant's confidence in filling out medical forms by themselves	Female: 1444<br>Male: 451	1408	404	99	Spanish	Not validated in the country of application	online questionnaire	General population
**Romero et al., 2018**	Brazil	175	70.73 ±7.64	1 - 4 years: 89<br>5 - 8 years: 66<br>> 8 years: 20	S-TOFHLA	Male+female: 175<br>Male: 64<br>Female: 111	40	66	69	Portuguese	Validated in the country	Households	Older people
**Rosas-Chavez et al., 2019**	Peru	276	18-29 years: 3<br>30-59 years: 82<br>>60 years: 91	Elementary school 116<br>Incomplete Elementary school 4<br>Incomplete high school 2<br>High school 133<br>Higher education 21	SAHLSA-50	Male+female: 276<br>Male: 92<br>Female: 184	177		99	Spanish	Validated in language but not country	Health services and others	Hypertensive patients
**Sá et al., 2022**	Brazil	150	18-39 years: 31<br>40-59 years: 74<br>>59 years: 45	Elementary school 70<br>High school 56<br>Higher education 24	S-TOFHLA	Male: 27<br>Female: 123	71	17	62	Portuguese	Validated in the country	Health services and other	Other pathologies

**Sampaio et al., 2015**	Brazil	82	42.4 ± 11.5	<8 years: 41	S-TOFHLA	Male+female: 82 Male: 19 Female: 63	28		54	Portuguese	Validated in the country	Health services and others	Diabetic patients
**Santos Júnior et al., 2021**	Brazil	62	60.4 ± 9.5	until 4 years: 5 5 to 8 years: 25 9 to 11 years: 21 12 years or more: 11	S-TOFHLA	Male: 16 Female: 46	31		31	Portuguese	Validated in the country	Health services and other	Diabetic patients
**Santos and Portella, 2016**	Brazil	114	67.4 ± 5.28	1 - 4 years:71 5 - 8 years:30 > 9 years:13	S-TOFHLA	Male+female: 114 Male: 41 Female: 73	18	12	84	Portuguese	Validated in the country	Health services and others	Older people
**Santos et al., 2017**	Brazil	63	66.21 ± 12.1	≤ 9 years: 40 > 9 years: 23	SAHLPA-50	Male+female: 63 Male: 30 Female: 33	32		31	Portuguese	Validated in the country	Health services and others	Heart disease patients
**Scortegagna et al., 2021**	Brazil	78	60-69 years: 47 70-79 years: 27 80-89 years: 4	1-4 years: 43 4-8 years: 24 >8 years: 11	S-TOFHLA	Male+female: 78 Male: 24 Female: 54	11	24	43	Portuguese	Validated in the country	Health services and others	Older people
**Silva et al., 2019**	Brazil	34	26 - 45 years: 8 46 - 59 years:12 > 60 years:14	Elementary school incomplete/complete 15 High school incomplete/co	S-TOFHLA	Male+female: 34 Male: 14 Female: 20	10	4	20	Portuguese	Validated in the country	Health services and others	Patients with nephropathies

				mplete 16 Higher education incomplete/complete 3									
**Silva et al., 2022**	Brazil	234	50 + 10.6	Elementary school 20 High School 102 Higher education 57 Post-graduation 55	S-TOFHLA	Female: 206 Male: 28	231	3		Portuguese	Validated in the country	web-based surveys	Hypertensive patients
**Silva-Junior et al., 2021**	Brazil	137	49–69 years: 71 23–48 years: 66	NR	HLS-14	Male+female: 137 Male: 37 Female: 100	77		60	Portuguese	Validated in the country	Households	General population
**Souza et al., 2014**[a]	Brazil	114	75.9 ± 6.2	4 (2–8) years	SAHLPA-18	Male+female: 129 Male: 38 Female: 76	56	0	58	Portuguese	Validated in the country	Health services and others	Older people
**Stonbraker et al., 2018**	Dominican Republic	107	40.8 ± 11.2	No formal education 12 Primary school or less 61 High school or less 28 All or some university 6	SAHL-S&E	Male+female: 107 (Transgender 2) Male: 41 Female: 64	33		74	Spanish	Validated in language but not country	Health services and others	HIV patients

**Tenani et al., 2021**	Brazil	238	62.7±10.55	Less than 4 years: 86<br>Up to 4 full years: 101<br>5 years or more: 51	HLS-14	Female: 165<br>Male: 73	69	85	84	Portuguese	Validated in the country	Health services and other	Health service users
**Torrejón-Peces et al., 2021**	Chile	215	72.7±5.9	No formal education 4<br>Basic 142<br>Medium 57<br>High 12	SAHLSA-50	Male: 63<br>Female: 152	171		44	Spanish	Validated in the country	Health services and other	Health service users
**Vílchez-Román et al., 2009**	Peru	49	27.7 ± 8.27	10.6 ± 2.32	SAHLSA-50	Male+female: 49<br>Male: 0<br>Female: 49	28		21	Spanish	Validated in language but not country	Health services and others	General population

Test of Functional Health Literacy in Adults (TOFHLA); Brazilian version of Short Test of Functional Health Literacy in Adults (S-TOFHLA); Short Assessment of Health Literacy for Portuguese-speaking Adults (SAHLPA); Short Assessment of Health Literacy for Spanish-speaking Adults (SAHLSA-50); Newest Vital Sign (NVS); Spanish version of the European Health Literacy questionnaire (HLS-EU-Q47); 18-item Short Assessment of Health Literacy Spanish and English (SAHL-S&E); 14-item Health Literacy Scale (HLS-14); Not reported (NR)

*Males and females were the total of participants and not only the participants evaluated for the HL: Abbott et al., 2018 (+8 participants); De Castro, 2014 (+14 participants); Diemer et al., 2017 (+1 participant); Ghisi et al., 2022 (-1 participant); Rivero-Méndez, 2015 (+1 participant) and Rodriguez et al., 2021 (-16 participants).

[a] 15 patients were considered illiterates if the SAHLPA-18 score was 0 or if individuals did not attempt to complete the test alleging being unable to read at all.
