# Supplementary material for: The low health literacy in Latin America and the Caribbean: a systematic review and meta-analysis
Source: BMC Public Health. 2024 Jun 1;24:1478. doi: 10.1186/s12889-024-18972-2 (PMC11144327; doi:10.1186/s12889-024-18972-2)
Supplement: Supplementary file 4 — Supplementary Material 4 [file 12889_2024_18972_MOESM4_ESM.pdf]

Additional file 4. Subgroup analyses.

Table 1. Subgroup analyses of reading and numeracy comprehension items.

Subgroup	N studies	N total	Prevalence [95%CI]	Prediction interval	I^2 (%)
**Target population**					
General population	7	6060	41.86 [30.75-53.87]	11.27-80.32	98.00
Health service users	7	2005	35.44 [24.74-47.82]	7.81-78.04	96.00
Hypertensive patients	3	825	29.01 [0.34-98.00]	0.00-100.00	93.00
Diabetic patients	6	656	36.07 [20.99-54.50]	3.48-89.82	94.00
Other pathologies	2	298	45.32 [37.65-53.22]	-	48.00
Older people	5	777	59.82 [46.93-71.48]	17.23-91.41	90.00
Heart disease patients	2	445	48.66 [16.58-81.89]	-	98.00
Children and adolescents	1	210	16.67 [11.89-22.41]	-	-
HIV patients	1	199	25.63 [19.72-32.28]	-	-
Patients with nephropathies	3	136	86.49 [40.14-98.39]	0.00-100.00	83.00
Caregivers and parents	1	80	15.00 [8.00-24.74]	-	-
**Country**					
Brazil	33	6399	46.28 [37.46-55.34]	9.36-87.79	96.00
Mexico	1	4044	30.51 [29.11-31.96]	-	-
Puerto Rico	2	950	41.82 [14.71-74.97]	-	98.00
Jamaica	1	88	27.27 [18.32-37.81]	-	-
Guatemala	1	210	16.67 [11.89-22.41]	-	-
**Setting**					
Health services	32	6338	45.39 [37.40-53.63]	10.98-84.85	96.00
Web-based surveys	2	4278	3.56 [0.02-87.09]	-	93.00
Households	3	865	60.97 [35.63-81.51]	0.00-100.00	98.00
Schools	1	210	16.67 [11.89-22.41]	-	-
**Tools**					
TOFHLA and S-TOFHLA	31	6059	45.40 [36.09-55.05]	8.46-88.21	96.00
NVS	7	5632	38.35 [24.74-54.06]	5.71-86.46	98.00
**Validity**					
Validated in the country	26	4990	46.63 [36.09-57.48]	8.10-89.65	96.00
Validated in language but not in the country	2	839	43.14 [16.52-74.42]	-	97.00
Validation study	4	1053	30.53 [20.27-43.17]	3.11-85.75	94.00
Not validated in the country of application	1	78	51.28 [39.69-62.77]	-	-
Not validated	5	4731	41.60 [19.35-67.89]	0.99-98.06	99.00

Table 2. Subgroup analyses of word recognition items.

Subgroup	N studies	N total	Prevalence [95%CI]	Prediction interval	I^2 (%)
**Target population**					
General population	4	506	44.59 [25.95-64.89]	1.50-97.70	94.00
Health service users	6	1959	47.44 [17.45-79.40]	0.46-99.44	99.00
Hypertensive patients	4	1373	58.15 [41.68-72.99]	5.23-97.22	97.00

Diabetic patients	2	331	38.16 [9.54-78.31]	-	98.00
Other pathologies	4	777	46.54 [24.93-69.53]	0.83-98.91	97.00
Older people	3	459	46.21 [23.02-71.17]	0.00-100.00	97.00
Heart disease patients	3	239	73.34 [48.68-88.86]	0.00-100.00	92.00
HIV patients	1	107	69.16 [59.50-77.73]		-
Patients with nephropathies	1	138	51.45 [42.80-60.04]		-
Caregivers and parents	1	125	59.20 [50.05-67.90]	-	-
**Country**					
Brazil	14	2864	65.71 [59.48-71.45]	39.66-84.82	87.00
Chile	4	1242	19.20 [16.33-22.44]	10.72-31.98	33.00
Peru	5	1160	36.07 [31.20-41.25]	21.53-53.71	64.00
Argentina	2	385	44.65 [19.05-73.44]	-	97.00
Dominican Republic	1	107	69.16 [59.50-77.73]	-	-
Barbados	1	106	19.81 [12.70-28.68]	-	-
Suriname	1	99	34.34 [25.09-44.56]	-	-
Costa Rica	1	51	100.00 [93.02-100.00]	-	-
**Setting**					
Health services	28	5895	51.69 [42.79-60.47]	13.11-88.35	97.00
Not reported	1	119	23.53 [16.24-32.18]		
**Tools**					
SAHLSA-50	10	2515	31.43 [23.69-40.37]	9.54-66.59	95.00
SAHLPA-18 and SAHLPA-50	13	2578	66.58 [60.12-72.47]	40.13-85.55	85.00
REALM, REALM-SF and REALM-D	3	491	35.49 [18.16-57.69]	0.00-100.00	95.00
SAHL-S&E	3	430	77.59 [15.37-98.51]	0.00-100.00	97.00
**Validity**					
Validated in the country	16	3594	54.28 [41.41-66.60]	10.63-92.22	98.00
Validated in language but not in the country	10	1545	43.02 [31.20-55.69]	10.44-83.02	92.00
Validation study	2	589	50.13 [21.87-78.30]	-	98.00
Not validated in the country of application	1	286	54.90 [48.93-60.76]	-	-

Table 3. Subgroup analyses of self-reported comprehension items.

Subgroup	N studies	N total	Prevalence [95%CI]	Prediction interval	I^2 (%)
**Target population**					
General population	4	2546	29.96 [9.95-62.34]	0.06-99.69	99.00
Health service users	4	1349	37.27 [27.04-48.84]	5.99-84.71	88.00
Diabetic patients	4	1008	35.54 [25.13-47.52]	5.51-83.91	90.00
Other pathologies	1	643	57.85 [53.93-61.70]	-	-
Heart disease patients	2	257	58.43 [49.92-66.46]	-	45.00
Children and adolescents	1	384	48.44 [43.34-53.56]	-	-
Caregivers and parents	1	22	86.36 [65.09-97.09].	-	-
**Country**					
Brazil	10	2182	43.90 [37.04-51.00]	21.87-68.63	83.00

Mexico	2	868	32.93 [15.19-57.37]	-	95.00
Puerto Rico	1	1911	5.18 [4.23-6.27]	-	-
Bolivia	1	643	57.85 [53.93-61.70]	-	-
Jamaica	1	355	48.17 [42.86-53.50]	-	-
Guyana	1	228	45.18 [38.60-51.88]	-	-
Honduras	1	22	86.36 [65.09-97.09]	-	-
**Setting**					
Health services	10	2337	44.13 [32.95-55.95]	11.92-82.17	96.00
Web-based surveys	2	2070	20.40 [1.22-84.20]	-	100.00
Households	4	1418	46.48 [43.89-49.08]	40.85-52.20	0.00
Schools	1	384	48.44 [43.34-53.56]	-	-
**Tools**					
Single Item	4	2880	36.27 [10.63-71.14]	0.03-99.92	100.00
HLS-14	3	518	41.29 [34.62-48.29]	3.13-93.87	61.00
Not reported	1	355	48.17 [42.86-53.50]	-	-
BRIEF	2	181	71.27 [33.17-92.54]	-	85.00
HLS-EU-Q47 and HLS-EU-Q6	4	1758	37.19 [26.35-49.49]	5.17-86.54	97.00
HLSQ	1	100	22.00 [14.33-31.39]	-	-
HLAT-8	1	33	42.42 [25.48-60.78]	-	-
10 self-reported questions	1	384	48.44 [43.34-53.56]	-	-
**Validity**					
Validated in the country	5	899	41.65 [35.98-47.56]	24.96-60.51	66.00
Validated in language but not in the country	3	1096	36.78 [22.73-53.50]	0.01-99.97	96.00
Validation study	2	926	46.22 [43.03-49.44]	-	0.00
Not validated in the country of application	6	2933	43.63 [17.39-73.99]	0.62-98.96	99.00
Not validated	1	355	48.17 [42.86-53.50]	-	-
