# Supplementary material for: The low health literacy in Latin America and the Caribbean: a systematic review and meta-analysis
Source: BMC Public Health. 2024 Jun 1;24:1478. doi: 10.1186/s12889-024-18972-2 (PMC11144327; doi:10.1186/s12889-024-18972-2)
Supplement: Supplementary file 5 — Supplementary Material 5 [file 12889_2024_18972_MOESM5_ESM.pdf]

Additional file 5. Sensitivity analysis.

Table 1. Sensitivity analysis of reading and numeracy comprehension items.

Excluded subgroup	N studies	N total	Prevalence [95%CI]	Prediction interval	I^2 (%)
Grey literature	32	10545	41.30 [33.03-50.08]	8.38-84.39	97.00
Children, adolescents, and older people	32	10704	42.52 [33.88-51.63]	8.20-85.96	97.00
Not validated tools and validation studies	29	5907	46.55 [37.17-56.17]	9.41-87.96	96.00
Health service users	31	9686	46.13 [36.58- 55.97]	8.33-88.98	97.00
Brazil	5	5292	30.91 [18.82-46.33]	3.32-85.36	98.00

Table 2. Sensitivity analysis of word recognition items.

Excluded subgroup	N studies	N total	Prevalence [95%CI]	Prediction interval	I^2 (%)
Grey literature	26	5551	50.69 [40.81-60.52]	10.96-89.57	98.00
Children, adolescents, and older people	26	5555	51.20 [41.66-60.66]	11.98-89.00	97.00
Not validated tools and validation studies	27	5425	50.71 [41.36-60.01]	11.84-88.74	97.00
Health service users	23	4055	52.90 [44.23-61.40]	16.18-86.72	96.00
Brazil	15	3150	34.93 [26.43-44.52]	9.06-74.32	94.00

Table 3. Sensitivity analysis of self-reported comprehension items.

Excluded subgroup	N studies	N total	Prevalence [95%CI]	Prediction interval	I^2 (%)
Grey literature	15	5071	41.08 [29.88-53.30]	7.74-85.29	98.00
Children, adolescents, and older people	16	5825	41.35 [30.82-52.74]	8.56-84.16	98.00
Not validated tools and validation studies	14	4928	40.79 [28.85-53.92]	6.91-86.47	98.00
Health service users	13	4860	43.58 [30.71-57.37]	7.33-88.30	99.00
Brazil	7	4027	40.24 [19.25-65.54]	1.49-96.78	99.00
