# Supplementary figures and images for: The low health literacy in Latin America and the Caribbean: a systematic review and meta-analysis
Source: BMC Public Health. 2024 Jun 1;24:1478. doi: 10.1186/s12889-024-18972-2 (PMC11144327; doi:10.1186/s12889-024-18972-2)

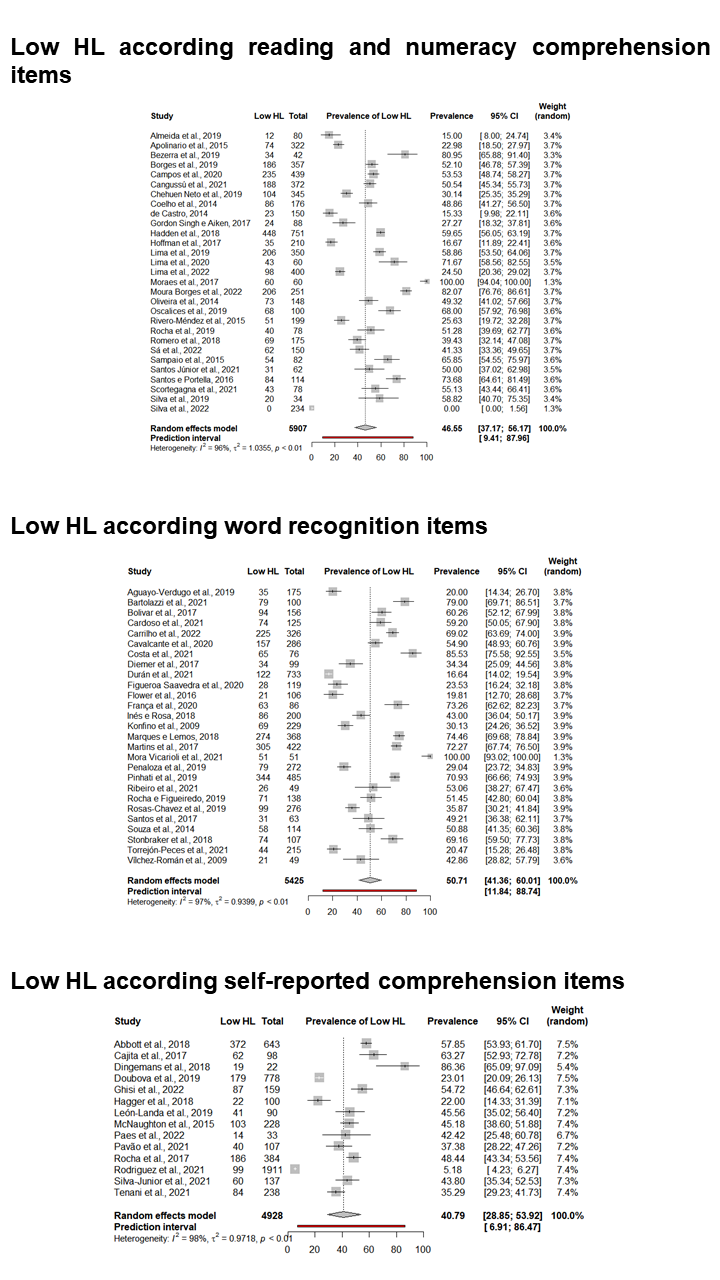

Supplement: Supplementary file 7 — Supplementary Material 7 [file 12889_2024_18972_MOESM7_ESM.png]

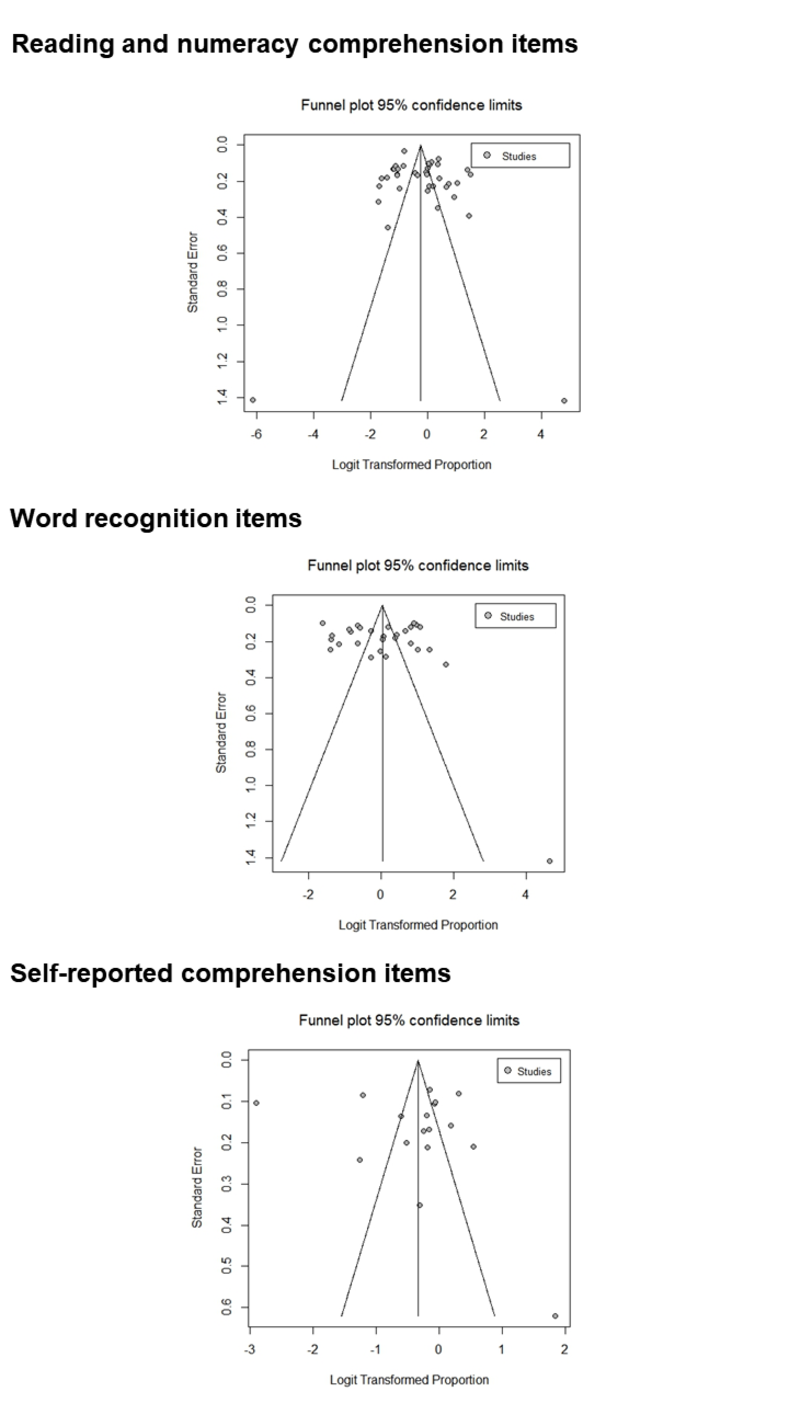

Supplement: Supplementary file 8 — Supplementary Material 8 [file 12889_2024_18972_MOESM8_ESM.tif]
